# Hormonal Contraceptive Formulations and Breast Cancer Risk in Adolescents and Premenopausal Women

**DOI:** 10.1001/jamaoncol.2025.4480

**Published:** 2025-10-30

**Authors:** Fatemeh Hadizadeh, Ardita Koteci, Torgny Karlsson, Weronica E. Ek, Åsa Johansson

**Affiliations:** 1 Department of Immunology, Genetics and Pathology, SciLifeLab, Uppsala University, Uppsala, Sweden

## Abstract

**Importance:**

Hormonal contraceptives are widely used but how breast cancer risk differs by hormonal content remains unclear.

**Objective:**

To estimate the difference in breast cancer risk associated with different hormonal contraceptive formulations.

**Design, Setting, and Participants:**

This Swedish nationwide, population-based cohort study was conducted using linked national registers. All adolescent girls and women aged 13 to 49 years residing in Sweden as of January 1, 2006, with no history of breast cancer, ovarian cancer, cervical cancer, uterine cancer, bilateral oophorectomy, or infertility treatment were included and followed up from 2006 to 2019. Individuals were censored on meeting an exclusion criterion, reaching age 50 years, or study end, whichever occurred first. Data were analyzed from November 2023 to August 2025.

**Exposure:**

Ever use and duration of use of hormonal contraceptives, categorized by hormone formulations and route of administration.

**Main Outcomes and Measures:**

Time-dependent Cox regression was used to estimate hazard ratios (HRs) with 95% CIs for incident cases of in situ and invasive breast cancer.

**Results:**

Among 2 095 130 adolescent girls and women (median [IQR] age at diagnosis, 45 [41-48] years) who were followed up for 21 020 846 person-years, 16 385 breast cancer cases occurred. Ever use of any hormonal contraceptive was associated with increased breast cancer risk (HR, 1.24; 95% CI, 1.20-1.28), corresponding to 1 additional case per 7752 (95% CI, 5350-14 070) users, with both combined (HR, 1.12; 95% CI, 1.07-1.17) and progestin-only formulations (HR, 1.21; 95% CI, 1.17-1.25) being associated. Higher risk was associated with oral desogestrel-only formulations (HR, 1.18; 95% CI, 1.13-1.23) and oral desogestrel-combined formulations (HR, 1.19; 95% CI, 1.08-1.31), as well as implants containing etonogestrel, desogestrel’s active metabolite (HR, 1.22; 95% CI, 1.11-1.35), compared to levonorgestrel-containing combined pills (HR, 1.09; 95% CI, 1.03-1.15) and levonorgestrel, 52 mg, intrauterine system (HR, 1.13; 95% CI, 1.09-1.18). No statistically significant increased risk was observed for medroxyprogesterone acetate injection, etonogestrel vaginal ring, or combined oral drospirenone, despite having many users.

**Conclusions and Relevance:**

Findings of this cohort study highlight that breast cancer risk varies substantially by progestin content in hormonal contraceptives, providing valuable insights to support more informed contraceptive prescription.

## Key Points

**Question:**

Does breast cancer risk differ by content of hormonal contraceptives?

**Findings:**

In this cohort study of more than 2 million adolescent girls and premenopausal women in Sweden, breast cancer risk varied by hormone formulation in hormonal contraceptives. Oral formulations containing desogestrel were associated with a higher number of additional cases per 100 000 person-years compared to those containing levonorgestrel.

**Meaning:**

These findings suggest that individuals at risk of breast cancer may benefit from avoiding desogestrel-containing hormonal contraceptives, particularly in progestin-only formulations.

## Teaser

This cohort study estimates the difference in breast cancer risk associated with different hormonal contraceptive formulations.

## Introduction

Breast cancer incidence is rising globally, particularly among premenopausal women,^1,2,3,4,5^ with worldwide projections estimating more than 3 million new cases and 1 million deaths annually by 2040.^6^ Hormonal contraceptives are a known risk factor,^7^ which, though conferring a modest risk at the individual level, translates into a substantial population-level impact given their widespread use.^8^ While estrogen’s role in promoting breast cancer through epithelial cell proliferation and oncogene amplification is well established,^9^ the role of progesterone and synthetic progestins is more debated,^10^ though some studies suggest they may also stimulate breast cell proliferation.^11,12^

Hormonal contraceptives consist of combined estrogen-progestin or progestin-only formulations and are administered orally or nonorally.^13^ Most existing studies have focused on combined oral contraceptives collectively,^14,15,16,17^ providing limited and often inconsistent evidence regarding individual progestins. Although a few large studies have examined a broader range of contraceptive options,^18,19,20^ their findings remain inconclusive, likely due to few users of certain products. An exception is the levonorgestrel intrauterine system (IUS), which has been investigated in 4 recent studies,^21,22,23,24^ though their findings are inconsistent and limited to this single formulation.

Leveraging high-quality nationwide Swedish register data, where progestin-only products are more prevalent than in most other countries, we formulated 2 hypotheses grounded in hormonal pharmacology. First, we hypothesized that progestin-only and combined contraceptives could have differential effects on breast cancer risk, as both hormone types can enhance breast epithelial cell proliferation^11,12^ and potentially promote carcinogenesis, while their combination may alter their metabolic processes and modify hormonal actions.^25,26^ Second, differences in progestin type could result in heterogeneity of risk, since synthetic progestins may be structurally related either to progesterone or to testosterone derivatives, with differences in potency and pharmacokinetics influencing systemic exposure and tissue effects.^27^ Additionally, progestins vary in binding affinities for progesterone, androgen, and glucocorticoid receptors,^27^ which determines their biological effects.^28^ We assessed these hypotheses in a cohort of more than 2 million adolescent girls and women of reproductive age, contributing more than 21 million person-years.

## Methods

### Study Population

We conducted a nationwide cohort study of all adolescent girls and women aged 13 to 49 years who were residing in Sweden as of January 1, 2006, using linked data from the Total Population Register and the Medical Birth, Patient, Education, Cancer, and Prescribed Drug Registers. Follow-up spanned from January 1, 2006, to ensure full-year data on hormonal contraceptive use from the Prescribed Drug Register, which began in July 2005, until December 31, 2019. Individuals with prior breast cancer, ovarian cancer, cervical cancer, uterine cancer, bilateral oophorectomy, or infertility treatment, or who died or emigrated before follow-up were excluded. Censoring occurred at age 50 years, meeting an exclusion criterion (eTable 1 in Supplement 1), or study end.

The study was approved by the Swedish Ethical Review Authority (Dnrs 2020-05348, 2021-05649-2, and 2024-07200-02), and individual informed consent was not required. We followed Strengthening the Reporting of Observational Studies in Epidemiology (STROBE) reporting guidelines.

### Hormonal Contraceptive Use

Hormonal contraceptive use was identified from the Prescribed Drug Register using Anatomical Therapeutic Chemical codes (eTable 2 in Supplement 1), capturing all redeemed prescriptions from 2006 to 2019. Contraceptive use was analyzed at 3 hierarchical levels: (1) any hormonal contraceptive, (2) main formulations (combined or progestin only), and (3) hormone formulations, including progestin type and route of administration. Emergency contraceptive pills were not included, as they are sold over the counter and not recorded in the register.

### Breast Cancer Ascertainment

Breast cancer cases were identified through the Swedish Cancer Register using *International Classification of Diseases for Oncology, Third Edition,* code C50, including both in situ and invasive cancers. Benign tumors were excluded (eTable 1 in Supplement 1).

### Covariates

Guided by a directed acyclic graph (eFigure 1 in Supplement 1), the main model included birth year; history of hysterectomy, unilateral oophorectomy, endometriosis, polycystic ovary syndrome, and sterilization; education level; number of childbirths; and hormonal contraceptive use in 2005 (only in duration and per milligram of use analyses). All covariates except birth year and prior contraceptive use were coded as time varying. Data on body mass index, smoking status, age at first birth, and previous contraceptive use (before 2006) were only available from the Medical Birth Register for 1 333 932 individuals (64% of the cohort) with a history of pregnancy; therefore, these variables were included as time-fixed covariates in a series of sensitivity analyses (eMethods and eTable 1 in Supplement 1). To assess the potential unmeasured confounding by variables that were not available in the registers, including early menarche, breastfeeding, and family history of breast cancer, we conducted a quantitative bias analysis (eMethods and eFigure 2 in Supplement 1).

### Statistical Analysis

We used age as the primary timescale and applied time-dependent Cox regression models to account for changes in exposures and covariates over time. Follow-up was split into multiple intervals per individual using the counting process format, enabling more accurate modeling of contraceptive use based on start and stop dates and allowing capture of switches between contraceptive types by treating these as separate intervals during an individual’s follow-up.^29^ This method accurately captures the time-varying exposure status and aims to avoid immortal time bias^30,31^ by classification of the treatment-free time before start of use as the unexposed follow-up time.^32^ This bias arises when a period during which the outcome cannot have occurred is incorrectly analyzed as time at risk. Hazard ratios (HRs) with 95% CIs were estimated for several exposure dimensions: (1) ever vs never use, (2) duration of use, and (3) progestin dose.

In the first analysis, exposure was dichotomized as ever vs never use of hormonal contraceptives. Individuals were classified as ever users from the date of first redemption and remained so thereafter. Those without any redemption were considered never users until first use or censoring. All individuals were considered unexposed at baseline unless they had already started in 2006, with exposure status updated on initiation.

To define duration of use, we calculated annual medication days based on pill count for oral products and the recommended duration for nonoral formulations (eMethods in Supplement 1). Duration of use was grouped into 4 intervals (<1 year, 1 to <5 years, 5-10 years, and >10 years), updated annually and treated as a categorical time-varying exposure. Nonlinear association between duration of use and breast cancer risk was assessed graphically by restricted cubic splines using the rms R package (R Project for Statistical Computing).

Current users and current plus recent users were defined from the first redemption date until 1 and 5 years after the last prescription of any type of hormonal contraceptive, respectively. To improve power and allow comparison with progestin-only counterparts, combined lynestrenol and norethisterone pills were grouped, as oral lynestrenol is rapidly converted to norethisterone in the liver.^33,34^

Both never/ever and duration of use analyses were applied to all 3 levels of hormonal contraceptives. We adjusted for the use of other contraceptive types during follow-up in each model, given that switching between products is common by users, with never users of each formulation of interest as the reference group.

When estimating breast cancer risk per milligram of progestin, annual dosage was calculated through multiplication of daily dose and annual medication days (eMethods in Supplement 1). For progestin dose analyses, HRs were estimated per milligram of progestin exposure for products with both combined and progestin-only formulations containing the same progestin, which limited the analyses to products containing desogestrel as well as lynestrenol and norethisterone. We further investigated the potential impact of estrogen dosage in combined formulations with desogestrel, for which multiple estrogen doses are available.

All hypothesis tests were 2-sided, and statistical significance was set at α = .05. We applied false discovery rate correction for multiple testing for each set of analyses, and a false discovery rate–adjusted *P* value of less than 0.05 was considered statistically significant. Comparisons between contraceptive methods with regard to magnitude of the HRs were limited to a selected set of methods, when no or minimal overlap between 95% CIs were detected, with *P* values not adjusted for multiple testing in these particular analyses. To estimate adjusted absolute risk and number needed to harm, we applied the Austin method^35,36^ (eMethods in Supplement 1).

All analyses were performed in R, version 4.3.1 (R Project for Statistical Computing). Data were analyzed from November 2023 to August 2025.

### Sensitivity Analyses

To assess the robustness of the findings, a series of sensitivity analyses were performed and are described in the eMethods in Supplement 1.

## Results

### Population Characteristics

**Figure 1.**
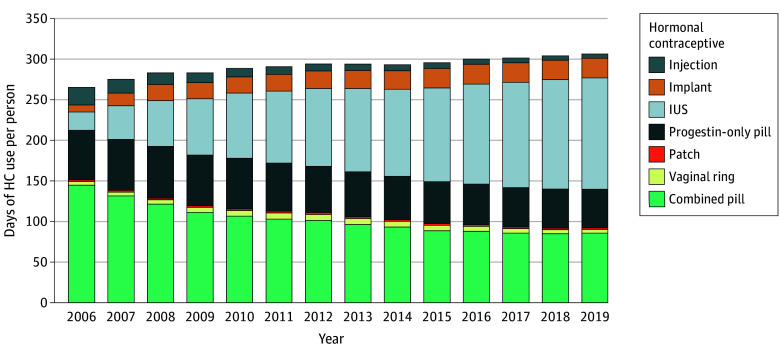
Patterns of Hormonal Contraceptive (HC) Prescriptions in Sweden During the Study Period Annual medication days of each year for each product was divided by the total number of adolescent girls or women using HC in that year. For intrauterine systems (IUSs) and implants, the duration of use was assumed to align with the recommended use for each product. However, devices prescribed before mid-2005 were not included, which may lead to an underestimation of their use in earlier years.

Between 2006 and 2019, the study accumulated 21 020 846 person-years and 16 385 incident breast cancer cases among 2 095 130 adolescent girls and women aged 13 to 49 years (mean [SD] follow-up, 10.03 [4.06] years). Of these, 1 284 613 individuals used hormonal contraceptives any time during follow-up (Figure 1), contributing 12 356 854 person-years and 8485 breast cancer cases. Those who never used hormonal contraceptives contributed 8 663 992 person-years and 7900 cancer cases. Median (IQR) age at diagnosis was 45 (41-48) years. Baseline characteristics are summarized in the Table and eTable 3 in Supplement 1.

**Table.** Basic Characteristics of Study Population (N = 2 095 130)

Characteristic	Person-years	Age, mean (SD), y^a^	Crude incidence rates^b^	Never pregnant, %^c^	No. of childbirths, median (IQR)	BMI, mean (SD)^d^	Education level, median (IQR)^e^	Ever smoked, %^d^
Never users of hormonal contraceptive	8 663 992^f^	31.98 (11.22)	91	31.4	1 (0-2)	24.12 (4.28)	3 (3-5)	19.5
Ever users								
Any type of hormonal contraceptive	12 356 854	28.8 (9.62)	69	26.5	1 (0-2)	24.20 (4.12)	3 (3-5)	19.2
Any type of combined hormonal contraceptive^g^	7 485 184	24.67 (7.48)	40	33.7	1 (0-2)	24.2 (4.12)	3 (3-5)	17.9
Any type of progestin-only hormonal contraceptive	8 144 294	31.58 (9.18)	89	21.5	1 (0-2)	24.51 (4.46)	3 (3-5)	21.6
Combined oral progestin								
Desogestrel	630 738	27.86 (7.05)	68	26.2	1 (0-2)	24.00 (3.91)	4 (3-5)	19.3
Levonorgestrel	4 339 546	23.59 (7.44)	34	36.7	1 (0-2)	24.29 (4.18)	3 (3-5)	18.5
Dienogest	144 059	28.46 (7.61)	49	39.4	0 (0-2)	23.73 (3.69)	4 (3-5)	16.8
Drospirenone	2 107 262	25.41 (6.58)	37	36.8	0 (0-2)	23.98 (3.92)	4 (3-5)	16.7
Nomegestrol	184 254	27.60 (6.45)	37	41.5	0 (0-2)	23.75 (3.61)	4 (3-5)	16.3
Norgestimate	898 874	22.02 (5.57)	19	41.4	0 (0-1)	24.07 (4.05)	3 (3-5)	17.9
Norethisterone/lynestrenol	775 063	25.21 (6.89)	44	30.3	1 (0-2)	24.04 (4.01)	3 (3-5)	16.1
Nonoral combined progestins								
Norelgestromin (patch)	263 218	24.86 (6.49)	38	29.7	1 (0-2)	24.49 (4.26)	3 (3-4)	27.4
Etonorgestrel (vaginal ring)	1 046 745	25.45 (5.98)	36	34.2	1 (0-2)	24.13 (3.89)	4 (3-5)	19.1
Progestin-only oral formulation								
Desogestrel	4 623 146	29.28 (8.66)	70	24.6	1 (0-2)	24.75 (4.65)	3 (3-5)	21.1
Levonorgestrel	80	35.86 (4.98)	0	0	1 (0-2)	25.45 (4.49)	3 (3-5)	28.6
Lynestrenol	529 650	33.15 (8.55)	104	16.9	2 (1-2)	24.17 (4.29)	3 (3-5)	18.8
Norethisterone	570 935	32.45 (8.62)	88	18.8	1 (0-2)	24.22 (4.25)	4 (3-5)	19.3
Progestin-only nonoral formulation								
Implant								
Etonogestrel	1 145 607	24.82 (7.45)	37	34.4	1 (0-2)	25.41 (4.90)	3 (3-4)	28.0
Levonorgestrel	32 693	26.99 (8.94)	61	25.0	1 (0-2)	25.28 (4.78)	3 (3-4)	26.7
Intrauterine system								
Levonorgestrel, 13.5 mg	98 900	29.43 (6.07)	50	38.4	1 (0-2)	24.33 (4.25)	4 (3-5)	16.6
Levonorgestrel, 19.5 mg	40 117	33.59 (6.61)	52	23.9	2 (1-2)	24.39 (4.28)	5 (3-5)	16.1
Levonorgestrel, 52 mg	2 777 293	35.91 (7.60)	128	11.5	2 (1-2)	24.37 (4.27)	4 (3-5)	18.7
Medroxyprogesterone acetate injection	640 529	35.60 (8.41)	108	21.1	2 (1-2)	24.85 (4.92)	3 (3-4)	32.7

Abbreviation: BMI, body mass index (calculated as weight in kilograms divided by height in meters squared).

^a^ Age at start of using contraceptives for users and age at baseline for never users.

^b^ No. of events per 100 000 person-years.

^c^ The percentage of adolescent girls and women who were never pregnant was calculated by dividing the number of individuals with no recorded pregnancy in the Medical Birth Register in each category by the total number of individuals in that category.

^d^ Smoking was defined as any smoking recorded at any prenatal visit; available only among women with a prior pregnancy. The percentage of those who smoke was calculated through dividing the number of individuals who smoke in each category by the number of individuals with history of pregnancy in that category.

^e^ Education level was recorded as (1) presecondary education shorter than 9 years, (2) presecondary education of 9 years, (3) secondary education, (4) postsecondary education shorter than 2 years, (5) postsecondary education of 2 years or longer, and (6) research training.

^f^ Indicates person-years for never users at baseline.

^g^ Combined hormonal contraceptives include ethinylestradiol except for Zoely (Anatomical Therapeutic Chemical code G03AA14) and Qlaira (Anatomical Therapeutic Chemical code G03AB08), which include estradiol hemihydrate and estradiol valerate, respectively.

### Ever Use of Hormonal Contraceptives

**Figure 2.**
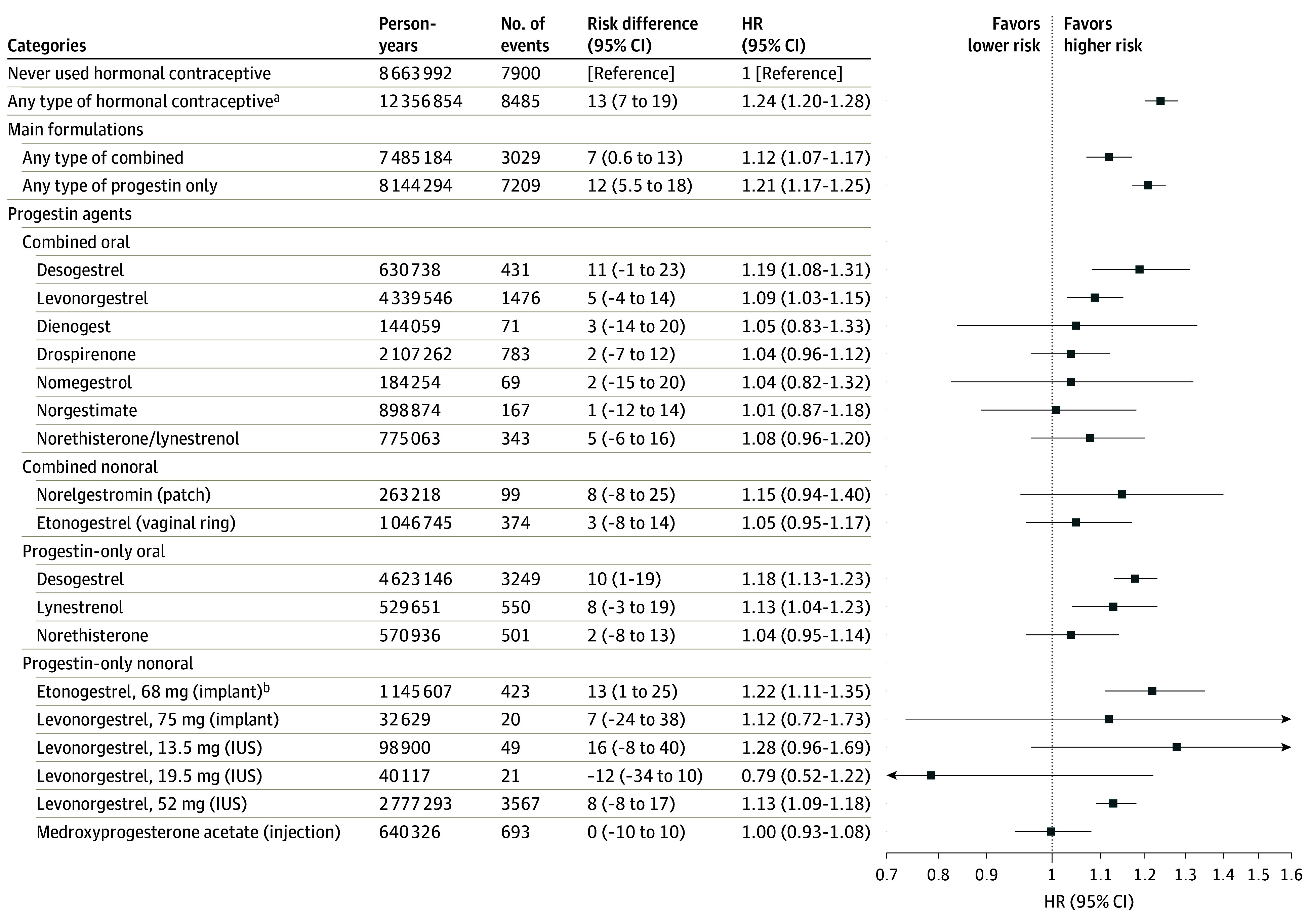
Breast Cancer Risk by Hormonal Contraceptive Type and Formulation Adjusted hazard ratios (HRs) for the use of different hormonal contraceptive formulations and progestin types on breast cancer incidence, relative to never users, are illustrated. The ever vs never use analyses were adjusted for birth year; history of hysterectomy, unilateral oophorectomy, endometriosis, polycystic ovary syndrome, and sterilization; education level; and number of childbirths. Risk difference indicates the overall absolute increase in breast cancers diagnosed among ever users of any hormonal contraceptive per 100 000 person-years. All initially statistically significant results remained significant after false discovery rate correction for multiple testing. ^a^The lower HR estimates observed in stratified analyses by main formulations, compared to analyses of any type, likely reflect frequent switching between products and the corresponding adjustment for use of other contraceptive types. ^b^Despite a numerically higher HR (1.22; 95% CI, 1.11-1.34) for etonogestrel implant, compared to most other methods, a very lower number of events was observed among implant users, explained by the younger average age at initiation compared to, for example, intrauterine system (IUS), 52 mg, users.

**Figure 3.**
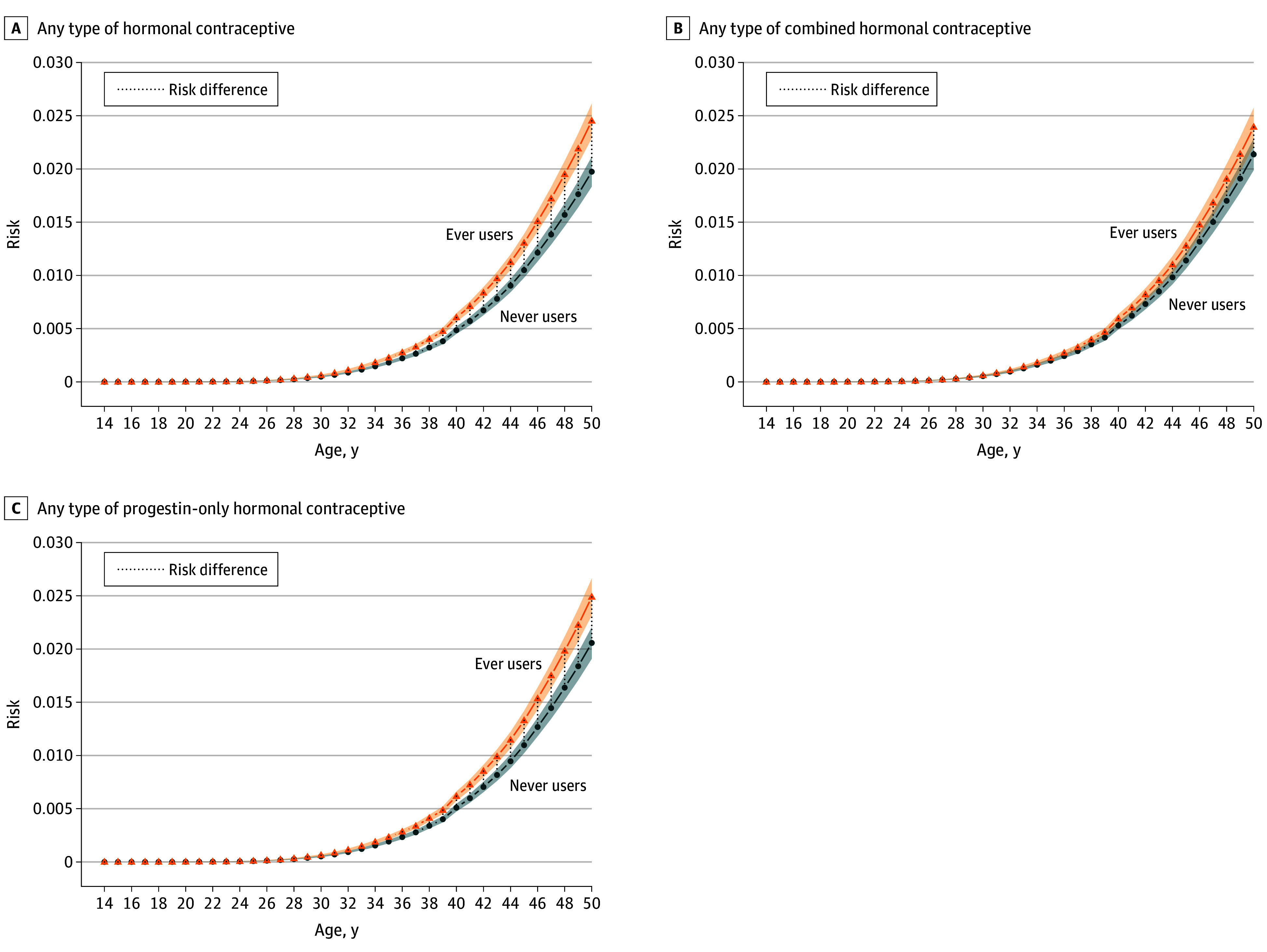
Absolute Risk of Breast Cancer Incidence by Age, Stratified by Hormonal Contraceptive Use The figure shows the adjusted absolute risk of breast cancer at different ages among users and never users of hormonal contraceptives. The analysis is further stratified into combined hormonal contraceptives and progestin-only contraceptives. The shaded areas represent 95% CIs. The risk differences reflect the estimated impact of hormonal contraceptive use on breast cancer risk across the age spectrum.

Ever use of any type of hormonal contraceptive was associated with an increased risk of breast cancer (HR, 1.24; 95% CI, 1.20-1.28; Figure 2), corresponding to 13 (95% CI, 7-19) additional cases per 100 000 person-years and 1 extra case per 7752 (95% CI, 5350-14 070) users per year (eTable 4 in Supplement 1). While both combined and progestin-only contraceptives were associated with increased breast cancer risk, there was a statistically significant greater risk for progestin-only contraceptives (HR, 1.21; 95% CI, 1.17-1.25) than combined methods (HR, 1.12; 1.07-1.17; Z test *P* = .006). This translated to 1 additional breast cancer case per 8572 users of progestin-only contraceptives compared to 14 417 for combined products. However, the median (IQR) duration of use was longer for progestin-only contraceptives (1350 [500-2635] days) than for combined (966 [364-1967] days), potentially contributing to the higher HR. The cumulative absolute risk in this cohort showed a steeper increase among older users (Figure 3).

### Ever Use of Different Progestin Agents

When further stratified by progestin type and route of administration, 6 contraceptive methods were statistically significant. Among these, desogestrel and etonogestrel, the active metabolite of desogestrel,^37,38^ were associated with higher risk than the products with levonorgestrel. For instance, HRs for oral desogestrel (combined: HR, 1.19; 95% CI, 1.08-1.31; progestin only: HR, 1.18; 95% CI, 1.13-1.23) and etonogestrel implants (HR, 1.22; 95% CI, 1.11-1.35) were higher than for oral combined levonorgestrel (HR, 1.09; 95% CI, 1.03-1.15) and levonorgestrel, 52 mg, IUS (HR, 1.13; 95% CI, 1.09-1.18). Progestin-only lynestrenol was also associated with increased breast cancer risk (HR, 1.13; 95% CI, 1.04-1.23). Other products, such as medroxyprogesterone acetate injection, etonogestrel vaginal ring, and combined oral drospirenone, despite having many users, were not associated with an increase in risk (Figure 2).

### Hormonal Contraceptives With Different Formulation Stratified by Duration of Use

**Figure 4.**
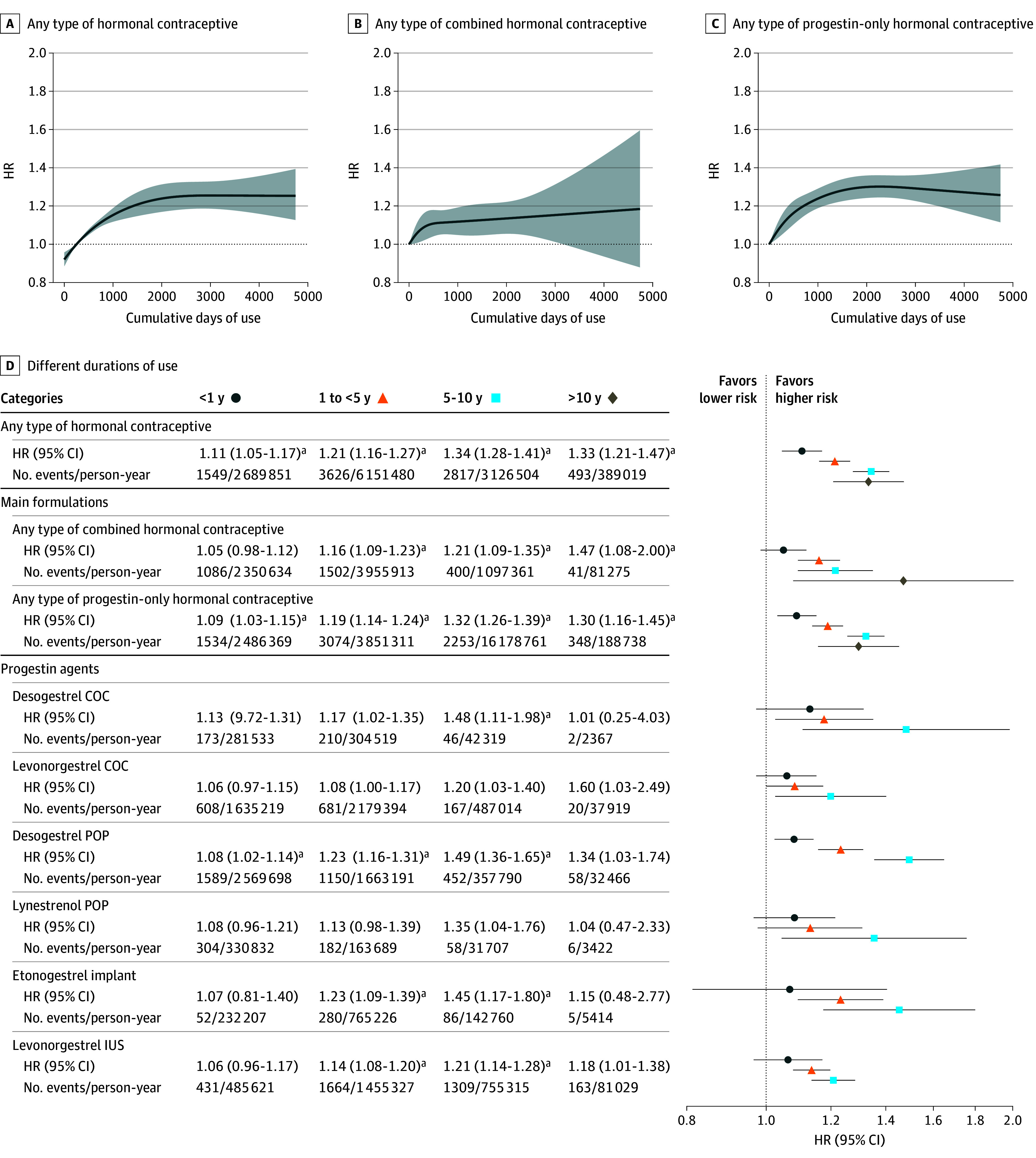
Association Between Duration of Use and Breast Cancer Risk A-C, Curves depict the nonlinear association between hormonal contraceptive exposure and breast cancer risk, modeled by restricted cubic splines (4 knots at equally spaced percentiles of different exposures). Curves for all progestin agents that were statistically significant in the ever vs never use analyses are available in eFigure 3 in Supplement 1. Shaded areas represent 95% CIs. D, Adjusted hazard ratios (HRs) and 95% CIs and corresponding forest plots for breast cancer associated with different durations of hormonal contraceptive use, stratified by main formulations and progestin agents. Only associations that were statistically significant in the ever vs never use analyses are illustrated. Results for all progestin agents are available in eTable 5 in Supplement 1. For progestin agents, estimates for durations more than 10 years are not illustrated due to wide confidence intervals, though all corresponding numerical values are reported. Adjusted HRs and 95% CIs from the rms::cph model (R Project for Statistical Computing) in restricted cubic spline are centered at the median of cumulative days of using hormonal contraceptives (reference, 252 days). All models are adjusted for birth year; history of hysterectomy, unilateral oophorectomy, endometriosis, polycystic ovary syndrome, and sterilization; education level; number of childbirths; and hormonal contraceptive use in 2005. COC indicates combined oral contraceptive; IUS, intrauterine system; POP, progestin-only pill. ^a^Statistically significant results after false discovery rate correction.

Longer duration of use was associated with a progressive increase in breast cancer risk (Figure 4 and eFigure 3 in Supplement 1). Less than 1 year of hormonal contraceptive use was associated with an HR of 1.11 (95% CI, 1.05-1.17). The HR increased with longer exposure, reaching 1.21 (95% CI, 1.16-1.27) for 1 to less than 5 years and 1.34 (95% CI, 1.28-1.41) for 5 to 10 years. In current users and current plus recent users, while short-term use (<1 year) was not statistically significant, HRs increased to 1.17 (95% CI, 1.12-1.23) and 1.21 (95% CI, 1.15-1.26) for 1 to less than 5 years and to 1.37 (95% CI, 1.30-1.44) and 1.36 (95% CI, 1.30-1.44) for 5 to 10 years, respectively (eTable 5 in Supplement 1). When examining the main formulations, there was not an association between short-term use of combined contraceptives and increased risk (HR, 1.05; 95% CI, 0.98-1.12), whereas there was an association with progestin-only products (HR, 1.09; 95% CI, 1.03-1.15).

Among progestins, only progestin-only oral desogestrel was associated with an increased risk after less than 1 year of use (HR, 1.08; 95% CI, 1.02-1.14). For 5 to 10 years of use, consistently elevated risks were observed across all desogestrel-containing formulations: progestin-only pills (HR, 1.49; 95% CI, 1.36-1.65), combined pills (HR, 1.48; 95% CI, 1.11-1.98), and the etonogestrel implant (HR, 1.45; 95% CI, 1.17-1.80). In comparison, the corresponding HRs for levonorgestrel combined pills and the levonorgestrel, 52 mg, IUS were 1.20 (95% CI, 1.03-1.40) and 1.21 (95% CI, 1.14-1.28), respectively (Figure 4 and eTable 5 in Supplement 1).

### Dose-Dependent Associations of Estrogen and Progestin With Breast Cancer Risk

Hormonal contraceptives vary in pharmacokinetics and potency, making milligram-based comparisons across progestins uninterpretable. However, some progestins are formulated both with and without estrogen, allowing estimation of the modifying effect by estrogen dose. Every additional milligram of unopposed oral desogestrel was associated with a higher HR (1.0021; 95% CI, 1.0017–1.0025) than estrogen-combined desogestrel (HR, 1.0011; 95% CI, 1.0005-1.0016; *P* = .002). In addition, while the merged category of norethisterone/lynestrenol-combined formulations showed no statistically significant association with breast cancer (HR, 1.0001; 0.9999-1.0002), each additional milligram of unopposed lynestrenol and norethisterone was associated with modestly increased HR at 1.0002 (95% CI, 1.0001-1.0004) and 1.0004 (95% CI, 1.0001-1.0006), respectively. To further investigate the potential modifying effect of estrogen in combined formulations, the risk associated with desogestrel combined with 20 μg vs 30 μg or more of ethinylestradiol was compared. Ever use of desogestrel combined with ethinylestradiol, 20 μg, was associated with a higher risk (HR, 1.33; 95% CI, 1.14-1.56) compared to the combined formulation at 30 μg or more (HR, 1.08; 95% CI, 0.95-1.22; *P* = .04), a pattern also reflected in dose-response analyses per milligram of desogestrel when combined with ethinylestradiol, 20 μg (HR, 1.0019; 95% CI, 1.0010-1.0027), vs 30 μg or more (HR, 1.0006; 95% CI, 0.9999-1.0013; *P* = .03).

### Sensitivity Analyses

All main findings were consistently supported by the extensive sensitivity analyses (eTables 6-8 and eFigures 2 and 4 in Supplement 1).

## Discussion

In this study, we confirmed that increased risk of breast cancer was associated with hormonal contraceptive use and translated to approximately 13 additional cases per 100 000 users per year. These findings echo those of older studies^14,15^ and align with more recent evidence,^18,19,20^ including 2 other Nordic register-based studies.^18,20^ Increased risk was evident for both combined and progestin-only products, with more pronounced results for progestin-only formulations. The widespread use of progestin-only and nonoral contraceptives in Sweden (Figure 1) enables comparisons rarely possible in other countries. For instance, regarding the etonogestrel-containing implant, we observed 1 145 607 person-years and 423 events among users compared to 42 217 person-years and 9 events in the most comprehensive study in Denmark.^18^ For the levonorgestrel IUS and progestin-only desogestrel, we included 2 777 293 and 4 623 146 person-years, respectively, far exceeding Denmark’s 503 441 and 77 847 person-years. This resulted in several important observations. Most notably, we found that levonorgestrel-based methods, both as combined oral pills and as IUS were associated with lower risk of breast cancer compared to products with desogestrel. These products were associated with only 5 (95% CI, –4 to 14) and 8 (95% CI, –8 to 17) additional breast cancer cases per 100 000 person-years, respectively. In contrast, desogestrel, which is widely used in Sweden in progestin-only, combined, and implant forms, was linked to higher risks, with 10 (95% CI, 1-19), 11 (95% CI, –1 to 23), and 13 (95% CI, 1-25) additional cases, respectively. This difference was also reflected in the duration of use analyses, particularly with 5 to 10 years of use. The present finding of increased breast cancer risk with levonorgestrel IUS aligns with recent evidence from register-based studies in Sweden,^21^ Denmark^23^ and Australia.^22^ Interestingly, these results point toward a relatively lower risk profile for combined drospirenone, as it was not associated with a statistically significant increase in risk (HR, 1.04; 95% CI, 0.96-1.12), despite its widespread use in Sweden, where we observed 2 107 262 person-years of follow-up, and similar results were reported in the previous Danish study.^18^ Additional products, such as medroxyprogesterone acetate injection, also appeared to be associated with lower or no increased risk. Collectively, results of this study suggest important differences in breast cancer risk between different progestin types.

These results also suggest that estrogen may attenuate progestin’s harmful effect. This is supported by 2 observations: (1) there were higher HRs per milligram of progestin in progestin-only pills compared to combined formulations containing the same progestin and (2) there were higher HRs per milligram of desogestrel in formulations with lower estrogen doses. Although we were unable to distinguish the effect of varying daily progestin doses, prior research suggests that reducing progestin dose alone does not proportionally reduce breast epithelial proliferation.^39^ While progestin-only products may confer higher risks per milligram due to the absence of estrogen, their lower progestin doses appear to balance the risk, and higher estrogen doses may potentially provide additional protective effects.

The finding that desogestrel may increase breast cancer risk more than other progestins is a novel finding that, to our knowledge, has not been previously reported. However, it is supported by underlying biological mechanisms. Progestins stimulate breast cancer cell proliferation mainly through binding to progesterone receptors.^40^ Progestins also bind to androgen receptors, whose signaling has antiproliferative and proapoptotic effects,^41^ and has even been explored as a therapeutic target in breast cancer.^42,43^ While desogestrel has slightly higher progesterone receptor affinity, it also shows considerably lower binding to androgen receptors^38^ compared to levonorgestrel,^44^ which agrees with findings in this study.

### Limitations

This study has several limitations. First, data on hormonal contraceptive use before mid-2005 were unavailable from the drug register, and misclassification of previous users as nonusers could have potentially attenuated the associations. However, history of contraceptive use, as well as data on body mass index, age at first birth, and smoking, were available for individuals with prior pregnancies, and sensitivity analyses indicated minimal residual confounding. Second, register data reflects prescriptions redeemed, not actual use, possibly biasing estimates toward the null. For long-acting reversible contraceptives such as IUS and implants, early discontinuations may have been missed, although pregnancy or method switching were used as proxies to identify this. Previous studies reported that 12% to 25% of women discontinue these methods within a year.^45,46,47^ In this cohort, 9.2% of IUS users and 12.4% of implant users discontinued within 1 year, suggesting minimal bias in estimated duration of use. Information on key possible confounders such as age at menarche, breastfeeding, and family history was unavailable. However, quantitative bias analyses showed that none of these unmeasured confounders altered the direction or statistical significance of the association, supporting the robustness of the findings. Also, while detection bias cannot be completely excluded, the risk difference remained statistically significant among women older than 40 years—when screening begins—suggesting the association is not solely driven by different access to early detection (Figure 3). Lastly, despite the large cohort, power was limited for less-commonly used progestins, warranting pooling of data in future international collaborations.

## Conclusions

This cohort study, to our knowledge, is the largest and most comprehensive study to date with extended follow-up and broad coverage of various progestin types. While the findings are robust, further research using causal inference methods and triangulation with other study designs is needed before clinical recommendations can be made. Although the relative risks are statistically significant, the absolute risk increase remains small and should be considered in the broader context of the well-established benefits of hormonal contraceptives. These benefits include prevention of unintended pregnancies, which are associated with increased maternal morbidity and mortality,^48^ as well as their protective effects against ovarian^49^ and endometrial^50^ cancers. Collectively, these considerations highlight the importance of personalized contraceptive counseling that takes into account individual risk profiles and preferences.

## Notes

Supplement 1.
eMethods

eReferences
**eTable 1.** Codes and information used to extract data from different registers **eTable 2.** Hormonal contraceptives and their corresponding progestin dosage and ATC codes **eTable 3.** Characteristics of study population **eTable 4.** Adjusted absolute risk, risk difference and crude proportions in users and never-users of hormonal contraceptives (HC) **eTable 5.** Duration of use analyses including all the progestin agents **eTable 6.** Sensitivity analyses for ever vs never users of different hormonal contraceptives (HC) **eTable 7.** Sensitivity analyses for duration of use analyses of different hormonal contraceptives **eTable 8.** Schoenfeld residual *P* values for all the main exposures and study covariates **eFigure 1.** Directed Acyclic Graph (DAG) on the association of hormonal contraceptive (HC) use and breast cancer **eFigure 2.** Quantitative bias analysis investigating the impact of binary unmeasured confounders of breast feeding, early menarche and family history of breast cancer on the estimated breast cancer hazard ratios in users of hormonal contraceptives **eFigure 3.** Restricted cubic spline curves for different progestin agents **eFigure 4.** Schoenfeld residual plot for covariate Birth year

Supplement 2.
Data Sharing Statement
