# Supplementary material for: Hormonal Contraceptive Formulations and Breast Cancer Risk in Adolescents and Premenopausal Women
Source: JAMA Oncol. 2025 Oct 30;11(12):1497–506. doi: 10.1001/jamaoncol.2025.4480 (PMC12576617; doi:10.1001/jamaoncol.2025.4480)
Supplement: Supplement 1. — eMethods eReferences eTable 1. Codes and information used to extract data from different registers eTable 2. Hormonal contraceptives and their corresponding progestin dosage and ATC codes eTable 3. Characteristics of study population eTable 4. Adjusted absolute risk, risk difference and crude proportions in users and never-users of hormonal contraceptives (HC) eTable 5. Duration of use analyses including all the progestin agents eTable 6. Sensitivity analyses for ever vs never users of different hormonal contraceptives (HC) eTable 7. Sensitivity analyses for duration of use analyses of different hormonal contraceptives eTable 8. Schoenfeld residual P values for all the main exposures and study covariates eFigure 1. Directed Acyclic Graph (DAG) on the association of hormonal contraceptive (HC) use and breast cancer eFigure 2. Quantitative bias analysis investigating the impact of binary unmeasured confounders of breast feeding, early menarche and family history of breast cancer on the estimated breast cancer hazard ratios in users of hormonal contraceptives eFigure 3. Restricted cubic spline curves for different progestin agents eFigure 4. Schoenfeld residual plot for covariate Birth year [file jamaoncol-e254480-s001.pdf]

Supplemental Online Content

Hadizadeh F, Koteci A, Karlsson T, Ek WE, Johansson Å. Hormonal contraceptive formulations and breast cancer risk in adolescents and premenopausal women. JAMA Oncol. Published online October 30, 2025. doi:10.1001/jamaoncol.2025.4480

eMethods
eReferences
eTable 1. Codes and information used to extract data from different registers
eTable 2. Hormonal contraceptives and their corresponding progestin dosage and ATC codes
eTable 3. Characteristics of study population
eTable 4. Adjusted absolute risk, risk difference and crude incidence rates in users and
never-users of hormonal contraceptives (HC)
eTable 5. Duration of use analyses including all the progestin agents
eTable 6. Sensitivity analyses for ever versus never users of different hormonal contraceptives (HC)
eTable 7. Sensitivity analyses for duration of use analyses of different hormonal contraceptives
eTable 8. Schoenfeld residual p-values for all the main exposures and study covariates
eFigure 1. Directed Acyclic Graph (DAG) on the association of hormonal contraceptive (HC) use and breast cancer
eFigure 2. Quantitative bias analysis investigating the impact of binary unmeasured confounders of breast feeding, early menarche and family history of breast cancer on the estimated breast cancer hazard ratios in users of hormonal contraceptives
eFigure 3. Restricted cubic spline curves for different progestin agents
eFigure 4. Schoenfeld residual plot for covariate Birth year

This supplemental material has been provided by the authors to give readers additional information about their work.

© 2025 Hadizadeh F et al. *JAMA Oncol*.

eMethods

Applied registry data

Information from six national registers was used in this study: 1) Medical Birth Register that includes data since 1973 on childbirth, and stillbirth with a gestational age of at least 22 weeks after 1 July 2008 and 28 weeks prior to that date, maternal height and weight during pregnancy (used to calculate BMI), and maternal smoking history at three time points—three months prior to pregnancy, at the first antenatal visit, and between gestational weeks 30 to 32. 2) Patient Register including inpatient register that contains information on all hospitalizations in Sweden, and outpatient register which provides records of all visits to healthcare facilities without hospital admission since 1997. 3) Swedish Cancer Register that tracks all cancer diagnoses in Sweden since 1958. 4) Swedish Prescribed Drug Register which offers data on all prescriptions redeemed at Swedish pharmacies from July 2005 onward, and 6) Swedish Education Register which provides annual updates on the education levels attained by all residents since 1985, with data reflecting the highest attained education level as of December 31 each year (eTable 1 in the Supplement). 7) Total Population Register, established in 1968 and maintained by Statistics Sweden, encompasses comprehensive demographic information, including data on births, migration, and deaths for all Swedish residents.

Hormonal Contraceptives

For oral contraceptives, the number of prescribed pills per visit was calculated by multiplying the Defined Daily Dose (DDD) of a medication by the prescription quantity (Fddd) (number of prescribed pills=Fddd × DDD). DDD is defined as the average daily dose when the drug is used by an adult for the drug's main indication. Fddd indicates how many DDD each prescription contains which itself is calculated through multiplication of the package's DDD by the number of prescribed packages. In case of missing information for oral preparations (e.g., ATC code G03AA16) data from forps column (presenting the number of tablets in each package) and the number of prescribed packages (from the ANTAL column) were used to calculate Fddd (Fddd = forps × ANTAL), and DDD was considered one. Due to lack of information for non-oral types of HC, calculation of Annual Medication Days (AMD) was performed based on information extracted from the FASS ("Farmaceutiska specialiteter i Sverige"; the main source of drug information in Sweden since 1966; https://www.fass.se) distinctively for each type of preparation.

The contraceptive vaginal ring, once inserted, should be left in place for 3 weeks (21 days) and the contraceptive patch is in place for one week (7 days). Therefore, AMD was estimated for vaginal rings and skin patches by multiplication of 21 and 7, respectively, to the number of prescriptions of the medication over the year (number of vaginal ring/patch in each package times the number of prescribed packages during each year).

© 2025 Hadizadeh F et al. *JAMA Oncol*.

Considering levonorgestrel-releasing intrauterine system (IUS), four different types of this preparation with different periods of use were prescribed in our study cohort. For the IUS, AMD was defined based on the type of IUS used by each woman (i.e., 3,5,6, and 8 years for Jaydess, Kyleena, Levosertone and Mirena, respectively). So, if a woman had redeemed a prescription for Jaydess on the April 1st 2008, she was considered to be exposed to the progesterone hormone for 270 days in 2008, 365 days in 2009 and 2010 and 95 days in 2010. The exposure to the hormone was set to end in case the user became pregnant or started to use another HC method. A similar approach was applied to sub-dermal implants, another long-acting progesterone-only method. For the implants the duration of exposure was considered 5 years for Norplant and Jadelle and 3 years for Implanon and its improved version Nexplanon. Although the participants have been followed from 1st January 2006, due to the long exposure to IUS and implants after insertion, the last six months of year 2005 was also considered when defining exposure to these two products. The progestin-only injectable product provides contraceptive effect for three months (91 days). Therefore, we calculated the AMD for injection by multiplication of 91 to the number of prescribed injections over a year.

Total exposure time was subsequently calculated as the cumulative number of days exposed to a full dose of the medication per year. For example, if a woman used an oral combined contraceptive for 120 days in 2007, 182 days in 2008, did not use in 2009, and was subsequently prescribed an implant 2 years before getting pregnant, her total exposure time was recorded as follows: 0 days in 2006, 120 days by the end of 2007, 302 days by the end of 2008, 302 days by the end of 2009, 667 days by the end of 2010, and 1032 days by the end of 2011, with the same number of exposed days persisting until the end of follow-up (2019).

For each product, we created a time-updated exposure variable representing initiation of that specific method. At baseline, all women are coded as non-users (value = 0) for all exposure variables. When a woman initiates use of, for example, a combined oral contraceptive (COC), the corresponding variable changes from 0 to 1 at the time of initiation and remains 1 for the rest of the follow-up, indicating "ever use." If she later switches to a progestin-only pill (POP), the POP variable similarly changes from 0 to 1 at the time of initiation. This structure allows us to adjust for use of other contraceptive methods in all analyses. In the ever/never analyses, once a woman has initiated a method, she is considered an "ever user" of that method for the remainder of follow-up.

In the duration analyses, each method is represented by a separate time-updated variable that accumulates exposure time. For example, a woman who alternates between COC and POP every six months over four years would accumulate two years of use for each method. In contrast, in the "any type" analysis, she would be counted as having four years of total hormonal contraceptive use.

Study Covariates

© 2025 Hadizadeh F et al. *JAMA Oncol*.

Smoking status was categorized as ever smoker if a woman reported smoking related to any of her pregnancies and never smoker if she did not. Due to the unavailability of data regarding the exact start time of smoking, this variable was treated as time-constant. A total of 1,218 women who reported to be smoker for only a short period of time (i.e., not smoking three months before pregnancy or at gestational weeks 30–32 but reported as smokers at the first antenatal visit) were excluded from the study. Body mass index (BMI) was calculated using weight and height data from the first pregnancy visit. For women with more than one pregnancy, the mean BMI across pregnancies was used.

The number of child births was dynamically updated based on new records in the registry data. For instance, if a woman already had two children in 2006, the live birth variable was coded as 2 at the start of study. When she had a third child, a new row was added to her data reflecting her age at the time of the third birth, and the live birth variable was updated to 3. Similarly, for a woman who had her first child in 2008, the live birth variable was coded as 0 in 2006 and 2007 and updated to 1 in 2008. Nulliparous women were consistently coded as 0 for this variable during the follow-up time.
To define previous contraceptive use, we used the column "Stopped taking birth control pills, date" from the Medical Birth Register. Women with any record in this column were classified as previous users of hormonal contraceptives. This variable was included as a time-constant binary variable (1 = yes, 0 = no) in the extended model among parous women.

The use of IUS in premenopausal women serves not only as contraception but also as a treatment for menorrhagia or heavy menstrual bleeding. While Mirena and Levosertone provide contraceptive protection for up to 8 and 6 years, respectively, their effectiveness for managing menorrhagia differs, with Mirena being effective for 5 years and Levosertone for 3 years. In this study, information regarding the specific indications for prescription was not available. Therefore, the duration of exposure to these two IUSs was considered to be 8 and 6 years, respectively, unless a pregnancy occurred or another type of contraceptive was initiated.

Statistical analysis

- Cox regression

We applied time-dependent Cox regression models using the "coxph" function in the "survival package" (version 3.5.5) in R (version 4.3.1). To incorporate time-varying covariates into the Cox proportional hazards model and estimate hazard rate ratios, the model for the hazard function λ, can be extended as follows:

$\lambda(t|X_i(t),Z_i) = \lambda0(t)\exp\ (\beta X_i(t) + \gamma Z_i)$
$HR= \lambda(t|X_i(t)=1,Z_i)/\ \lambda(t|X_i(t)=0,Z_i) = \lambda0(t)\exp(\beta*1+ \gamma Z_i)/\lambda0(t)\exp(\beta*0+ \gamma Z_i) = \exp(\beta)$

© 2025 Hadizadeh F et al. *JAMA Oncol*.

In these expressions, $\lambda 0(t)$ represents the baseline hazard function, while β and γ denote the coefficients corresponding to the time-varying ($X_i(t)$) and time-fixed (Z_i) covariates, respectively. The time-varying variable, X, is permitted to change over time for different individuals *i*, while the associated effect β of X is considered time-constant.

- Assessment of the Proportional Hazards Assumption

We evaluated the proportional hazards assumption using the Schoenfeld residuals test. All main exposures satisfied the assumption ($p > 0.05$), after FDR correction for multiple testing (eTable 8 in the Supplement), except for the variable "birth year". However, inspection of the corresponding Schoenfeld residual plots did not suggest any substantial deviations from proportionality (eFigure 4 in the Supplement). To explore this further, we also conducted a sensitivity analysis excluding birth year from the models. The results showed only minor changes in the effect estimates, with no shift in statistical significance or direction of effect.

- Risk difference

To estimate the risk difference (absolute risk increase) and the number needed to harm (NNH), we applied the method developed by Austin for estimation of risk difference in survival analysis.[1] For this analysis, a Cox proportional hazards model was fitted using the cph() function from the "rms" R package.[2] The model was adjusted for birth year, endometriosis, unilateral oophorectomy, hysterectomy, polycystic ovary syndrome, sterilization and education as covariates. Confidence intervals were calculated using Altman's method. In this method, confidence intervals are calculated based on the standard errors of survival probabilities for the exposed and unexposed groups. These were obtained using the "survest" function from the "rms" package in R[3].

- Pairwise test difference

To test for differences between hormonal contraceptives, we calculated p-values for pairwise comparisons when confidence intervals showed minimal or no overlap. Standard errors and covariances between pairs of effect estimates were adopted from the vcov() function, which estimates the variance-covariance matrix.

Risk of breast cancer in ever users of hormonal contraceptives versus never users

We employed different models to estimate the risk of exposure to HCs at various levels of detail. To simulate real-world HC use where a woman may be exposed to various types of hormones throughout her life, and to account for transition and shifting between

© 2025 Hadizadeh F et al. *JAMA Oncol*.

different hormonal products, we included exposure to all types of HC within a single model. Therefore, the risk associated with each specific preparation compared to never users is estimated by adjusting for exposure to all other preparations over the follow-up. That means, we included all different routes of administration of oral combined, oral progestin-only, IUS, implant, injection, patch and vaginal ring in one model. Stratifying by progestin type and dosage enabled a detailed investigation of the association between hormonal contraceptive use and breast cancer risk. In this analysis, we included four different types of oral progestin-only (desogestrel, levonorgestrel, lynestrenol, and norethisterone), seven types of oral combined formulations (desogestrel, levonorgestrel, norethisterone/lynestrenol (merged), dienogest, drospirenone, nomegestrol, and norgestimate) (for further information see Supplementary eTable 2). In addition, implants based on the content and dosage of their progestin were categorized in three types: 36 and 75 milligrams (mg) of levonorgestrel and one type containing 68mg of etonogestrel. IUS was also categorized in three different groups: 13.5mg for three years (jaydess), 19.5 mg for five years (kyleena) and 52mg for 6 to 8 years (levosertone and mirena) of levonorgestrel.

Due to the limited number of women using combined HCs containing the first generation progestins lynestrenol and norethisterone, given these two items are deregistered in Sweden, and considering that lynestrenol is a prodrug that rapidly and almost completely converts into norethisterone in the liver upon oral administration, [4–6] these two progestins were combined into a single group for analysis to attain a comparable power with their corresponding progestin-only products.

Dosage calculation

Information on the progestin dosage of each hormonal contraceptive was primarily obtained from the "styrkalf" column in the Prescribed Drug Register. For missing data, additional information was retrieved from the FASS website. In cases of deregistered medications, dosage data were sourced from the Swedish Medical Products Agency (www.lakemedelsverket.se).
For dose calculations of long acting products, since hormone release decreases over time and duration of use varies among women, the average release per 24h over the first year was obtained from the FASS website.

Sensitivity analyses

To validate our results, we conducted a series of sensitivity analyses (eTable 6 and eTable 7 in the Supplement).

First, we evaluated the impact of adjusting for key confounders of age at first birth, prior use of hormonal contraceptives, body mass index (BMI), and smoking status, which were available only for parous women through the National Medical Birth Register. These variables were incorporated into a set of extended models. To evaluate the specific

© 2025 Hadizadeh F et al. *JAMA Oncol*.

contribution of these variables, results from the extended models were compared with those from models applied to the same subgroup but without adjustment for these variables.

Second, in cases where the calculated AMD based on the Prescribed Drug Register data exceeded the number of days in a year, a maximum limit of 365 days per year was applied to represent full exposure to HC, and the values above 365 days were rounded down to 365 days. Sensitivity analyses were conducted to assess the impact of limiting the number of days of using HC to 365 days per year (365 days limited analyses) on the estimated effect.

Finally, to capture all cancer cases recorded in either the cancer register or the patient register, we conducted a sensitivity analysis by supplementing the cancer register data with cases identified in the inpatient register using ICD-10 codes "C50" and "D05" (referred to as the "all sources cancer" analysis). In cases of overlapping records, the earliest year documented in either register was used to determine the age at cancer diagnosis.

Quantitative Bias Analysis

Given the potential for unmeasured confounding, we conducted a quantitative bias analysis (QBA)[7] to obtain bias-adjusted hazard ratios (HRs) for three binary confounders: early menarche, breastfeeding, and positive family history of breast cancer. The QBA required specifying the following parameters: the prevalence of each confounder among hormonal contraceptive users and never users, and the estimated HR for the association between each confounder and breast cancer.

We performed a literature review and extracted three HR estimates for each confounder's association with breast cancer. The prevalence of each confounder among never users was based on general population data and held constant. Prevalence among users was estimated using published evidence or informed assumptions. For each confounder, we defined nine scenarios to estimate the potential impact on our study results.

An exception was made for family history of breast cancer. Although women with a positive family history are generally expected to use hormonal contraceptives less frequently, Mørch et al. reported slightly higher use in this group[8]. Therefore, we considered 18 scenarios representing both lower and higher prevalence among users.

The bias factor on the HR scale was calculated using the formula:

Bias factor=[1 + (HR − 1)p1]/[1 + (HR − 1)p0]
where *HR* is the confounder–breast cancer association, p_1 is the confounder prevalence among ever users, and p_0 is the prevalence among never users[9].

© 2025 Hadizadeh F et al. *JAMA Oncol*.

The bias-adjusted HR (the estimate we would have obtained if we had controlled for the unmeasured confounder) was then obtained by dividing the observed study HR by the calculated bias factor.

In quantitative bias analysis, across all hypothetical scenarios tested, none altered the direction or statistical significance of the association, indicating that the main finding was robust to a range of plausible unmeasured confounding (eFigure 2 in the Supplement).

Accounting for Induction and Latency Periods in cancer development

Current evidence suggests that for development of cancer, there is a time interval comprising the induction phase, from causal trigger to disease onset, and a latency phase, from onset to detection, with the duration of this interval depending on the nature of the exposures[10]. As the length of these intervals is often uncertain, it has been suggested that examining multiple durations after initiation of exposure should be considered in cancer epidemiology studies[11]. Therefore, we applied an alternative coding approach wherein HRs for breast cancer risk were estimated for specific time intervals following HC initiation: within one year, two to three years, four to five years, and more than five years and estimated HRs for these specific time intervals following HC initiation. In the first year after starting HC, based on 1,282,228 person-years and 593 events, the adjusted HR was 1.10 (95% CI: 1.01 to 1.20). During the second-third years, with 2,460,768 person-years and 1,317 events, the HR was 1.16 (95% CI: 1.10 to 1.23). For the fourth-fifth years, based on 2,290,598 person-years and 1,421 events, the HR was at 1.22 (95% CI: 1.15 to 1.29). Finally, beyond five years after initiation, the HR increased to 1.32 (95% CI: 1.26 to 1.38), based on 6,323,260 person-years and 5,154 events.

eReferences

1. Austin PC. Absolute risk reductions and numbers needed to treat can be obtained from adjusted survival models for time-to-event outcomes. *Journal of Clinical Epidemiology*. 2010;63(1):46-55. doi:10.1016/j.jclinepi.2009.03.012

2. Harrell Jr FE, Harrell Jr MFE, Hmisc D. Package 'rms.' *Vanderbilt University*. 2017;229(201):7.

3. Zhang Z, Ambrogi F, Bokov AF, Gu H, de Beurs E, Eskaf K. Estimate risk difference and number needed to treat in survival analysis. *Ann Transl Med*. 2018;6(7):120. doi:10.21037/atm.2018.01.36

4. Odlind V, Weiner E, Victor A, Johansson EDB. Plasma Levels of Norethindrone After Single Oral Dose Administration of Norethindrone and Lynestrenol. *Clinical Endocrinology*. 1979;10(1):29-38. doi:10.1111/j.1365-2265.1979.tb03030.x

5. Kuhl H, Bremser HJ, Taubert HD. Serum levels and pharmacokinetics of norethisterone after ingestion of lynestrenol: Its relation to dose and stage of the menstrual cycle. *Contraception*. 1982;26(3):303-315. doi:10.1016/0010-7824(82)90078-6

6. Mazaheri A, Fotherby K, Chapman JR. METABOLISM OF LYNESTRENOL TO NORETHISTERONE BY LIVER HOMOGENATE. *Journal of Endocrinology*. 1970;47(2):251-252. doi:10.1677/joe.0.0470251

7. Lash TL, Fox MP, MacLehose RF, Maldonado G, McCandless LC, Greenland S. Good practices for quantitative bias analysis. *Int J Epidemiol*. 2014;43(6):1969-1985. doi:10.1093/ije/dyu149

8. Mørch LS, Skovlund CW, Hannaford PC, Iversen L, Fielding S, Lidegaard Ø. Contemporary Hormonal Contraception and the Risk of Breast Cancer. *N Engl J Med*. 2017;377(23):2228-2239. doi:10.1056/NEJMoa1700732

9. Collaborative TO, Tazare J, Nab L, et al. Effectiveness of Sotrovimab and Molnupiravir in community settings in England across the Omicron BA.1 and BA.2 sublineages: emulated target trials using the OpenSAFELY platform. *medRxiv*. Preprint posted online May 16, 2023:2023.05.12.23289914. doi:10.1101/2023.05.12.23289914

10. Rothman KJ. INDUCTION AND LATENT PERIODS. *American Journal of Epidemiology*. 1981;114(2):253-259. doi:10.1093/oxfordjournals.aje.a113189

11. Hicks B, Kaye JA, Azoulay L, Kristensen KB, Habel LA, Pottegård A. The application of lag times in cancer pharmacoepidemiology: a narrative review. *Annals of Epidemiology*. 2023;84:25-32. doi:10.1016/j.annepidem.2023.05.004

© 2025 Hadizadeh F et al. *JAMA Oncol*.

eTable 1. Codes and information used to extract data from different registers.

Category	Code	Source	Description
Outcome			
Breast Cancer; main analysis	ICDO3* codes of C500:C509	Cancer register	SNOMED3 code of 90201 was used to extract benign tumours to exclude from the main analysis
Breast Cancer; All sources cancer sensitivity analyses	ICDO3 codes of C500:C509	Cancer register	Inclusion of all breast cancer codes, including codes for possible benign cases
	ICD-10 codes of C50, C50-, C500:C506, C508, C509, C509M and D05	Inpatient and outpatient registers	
Excluding/censoring variables			
History of Breast cancer	ICD7* codes of 1701, 1702, 1707, 1708, 1709	Cancer register	
Ovarian cancer	ICD7 codes of 1750, 1751, 1759, 1769, 1994, 1758<br>ICD10 codes of C569: C579	Cancer register	
Endometrial cancer	ICD7 codes of 172, 174	Cancer register	
Cervical cancer	ICD7 code of 171	Cancer register	
Bilateral oophorectomy	LAE20, LAE21, LAF10, LAF11	Inpatient register	Including bilateral oophorectomy and bilateral salpingo-oophorectomy
History of using infertility medication	ATC codes of G03GA, G03GB and L02	Prescribed Drug Register	
Adjusting covariates			
Endometriosis	N80	Inpatient register	
polycystic ovary syndrome	E282, L680	Inpatient register	
Hysterectomy	LCD00-97	Inpatient register	
Unilateral oophorectomy	LAE10-11, LAF00-01	Inpatient register	Including unilateral oophorectomy and unilateral salpingo-oophorectomy
Sterilisation	LGA00<br>LGA10,11	Inpatient register	

© 2025 Hadizadeh F et al. *JAMA Oncol*.

	LGA20-22<br>LGA96-98		
Education	-	The education register	Coded as:<br>1) Pre-secondary education shorter than 9 years<br>2) Pre-secondary education 9 years (equivalent)<br>3) Secondary education<br>4) Post-secondary education shorter than two years<br>5) Post-secondary education two years or longer<br>6) Research training<br>9) Data missing
Number of live births, smoking, weight and height (to calculate Body Mass Index; BMI)	-	National Medical Birth Register	It covers all newborns as well as stillbirths with a gestational age of at least 28 weeks (before July 2008) and 22 weeks (after July 2008).<br>Data for smoking was available for:<br>- 3 months before first antenatal visit<br>- Day of the first antenatal visit<br>- Week 30-32<br>Data for weight and height was available from the first antenatal visit.

***** ICD(International Classification of Diseases)-O/3, the most updated code applied from 2005, was used to extract main outcome.

ICD7, the most comprehensive code started from 1958, was employed as the main source code to extract patients for exclusion and censoring. Other ICD codes, including ICD9 (from 1987), ICD-O/2 (from 1993) and ICD-O/3 (from 2005) were also searched and in case of difference, the patients were added to those extracted from ICD7 codes.

For extraction of cancer cases, all the columns related to main diagnosis and other diagnoses were explored.

© 2025 Hadizadeh F et al. *JAMA Oncol*.

eTable 2. Hormonal contraceptives and their corresponding progestin dosage and ATC codes

Hormonal contraceptives	ATC* codes	Hormone dosage (mg)
Combined		
**Oral**		
Desogestrel	G03AA09, G03AB05	0.15, 0.1
Levonorgestrel	G03AA07, G03AB03	0.15, 0.10, 0.083
Norgestimate	G03AA11	0.25
Norethisterone	G03AA05, G03AB04	0.5, 0.75
Lynestrenol	G03AA03	0.75
Drospirenone	G03AA12	3
Nomegestrol	G03AA14	2.5
Dienogest	G03AA16, G03AB08	2
**Non oral**		
Patch		
Norelgestromin	G03AA13	6 (0.203mg/24h)
Vaginal ring		
Etonogestrel	G02BB01	11.7 (0.120 mg/24h)
Progestin-only		
**Oral**		
Desogestrel	G03AC09	0.075
Levonorgestrel	G03AC03	0.03
Norethisterone	G03AC01	0.35
Lynestrenol	G03AC02	0.5
**Non oral**		
Implant		
Etonogestrel	G03AC08	68 (0.04mg/24h)
Levonorgestrel	G03AC03	36 (0.02mg/24h)
Levonorgestrel	G03AC03	75 (0.041mg/24h)
IUS		
Levonorgestrel	G02BA03	13.5 (0.008mg/24h)<br>19.5 (0.013mg/24h)<br>52 (0.02mg/24h)
Injection		
Medroxyprogesterone acetate	G03AC06	150mg/ml #

* ATC = Anatomical Therapeutic Chemical classification
# Each injection consists of 1ml and is effective for three months
Combined hormonal contraceptives include ethinyl estradiol except for Zoely (ATC code: G03AA14) and Qlaira (ATC code: G03AB08) that include estradiol hemihydrate and estradiol valerate, respectively.

© 2025 Hadizadeh F et al. *JAMA Oncol*.

eTable 3. Characteristics of study population

	Person years	Age at baseline (2006)	Age at start of using HC	Education[a] Median (IQR)	Nulli gravid %	BMI[b] (mean±sd)	Smoking[b] %	Endometriosis %	Unilateral oophorec tomy %	Hysterectomy %	PCOS %	Sterilization %	No. of child birth Median (IQR)
Never users of HC	8663992	31.98±11.22	-	3 (3-5)	31.42	24.12 ± 4.28	19.47	0.54	0.38	0.75	0.02	0.46	1 (0-2)
Ever users of													
Any type of HC	12356854	26.96±9.58	28.8±9.62	3 (3-5)	26.47	24.20 ± 4.12	19.19	0.86	0.50	0.95	0.08	0.31	1 (0-2)
Combined HC	7485184	22.83±7.71	24.67±7.48	3 (3-5)	33.70	24.2±4.12	17.85	0.80	0.38	0.50	0.09	0.21	1 (0-2)
Progestin-only HC	8144294	28.32±9.66	31.58±9.18	3 (3-5)	21.51	24.51±4.46	21.62	0.92	0.55	1.12	0.07	0.36	1 (0-2)
**Combined oral progestins**													
Desogestrel	630738	26.62±7.45	27.86±7.05	4 (3-5)	26.21	24.00±3.91	19.27	1.08	0.49	0.50	0.21	0.22	1 (0-2)
Levonorgestrel	4339546	21.54±7.63	23.59±7.44	3 (3-5)	36.66	24.29±4.18	18.52	0.83	0.36	0.44	0.06	0.19	1 (0-2)
Dienogest	144059	21.49 ± 7.45	28.46 ± 7.61	4 (3-5)	39.38	23.73±3.69	16.78	2.33	0.47	0.96	0.13	0.21	0 (0-2)
Drospirenone	2107262	21.71±6.95	25.41±6.58	4 (3-5)	36.81	23.98±3.92	16.72	0.86	0.36	0.43	0.17	0.18	0 (0-2)
Nomegestrol	184254	20.06±6.29	27.60±6.45	4 (3-5)	41.47	23.75±3.61	16.31	0.94	0.26	0.41	0.08	0.10	0 (0-2)
Norgestimate	898874	19.21±5.52	22.02±5.57	3 (3-5)	41.37	24.07±4.05	17.91	0.48	0.24	0.18	0.08	0.10	0 (0-1)
Norethisterone/ Lynestrenol	775063	23.49±7.51	25.21±6.89	3 (3-5)	30.32	24.04±4.01	16.13	0.57	0.33	0.54	0.05	0.19	1 (0-2)
**Nonoral combined progestins**													
Norelgestromin	263218	21.06 ± 6.80	24.86±6.49	3 (3-4)	29.69	24.49±4.26	27.37	0.89	0.27	0.50	0.07	0.41	1 (0-2)
Etonogestrel	1046745	21.12±6.46	25.45±5.98	4 (3-5)	34.20	24.13±3.89	19.14	0.70	0.29	0.34	0.08	0.20	1 (0-2)
**Progestin-only oral formulation**													
Desogestrel	4623146	25.77±8.78	29.28±8.66	3 (3-5)	24.62	24.75±4.65	21.14	0.97	0.50	0.83	0.07	0.35	1 (0-2)
Levonorgestrel	80	35.29±4.68	35.86±4.98	3 (3-5)	0	25.45±4.49	28.57	0	0	0	0	0	1 (0-2)
Lynestrenol	529650	30.34±9.23	33.15±8.55	3 (3-5)	16.85	24.17±4.29	18.76	0.83	0.53	1.02	0.06	0.43	2 (1-2)

© 2025 Hadizadeh F et al. *JAMA Oncol*.

Norethisterone	570935	28.69±9.19	32.45±8.62	4 (3-5)	18.77	24.22±4.25	19.27	0.81	0.50	1.00	0.07	0.37	1 (0-2)
**Progestin-only nonoral formulation**													
**Implant**													
Levonorgestrel[c]	235	33.35±7.67	36.46±8.76	3 (3-3)	11.54	25.05±5.32	43.48	0	3.85	3.85	0	0	2 (1-3)
Etonogestrel[d]	1145607	20.88±7.21	24.82±7.45	3 (3-4)	34.36	25.41±4.90	28.02	0.50	0.31	0.40	0.09	0.28	1 (0-2)
Levonorgestrel[e]	32693	26.14±8.94	26.99±8.94	3 (3-4)	24.98	25.28±4.78	26.69	0.79	0.49	1.28	0.07	0.66	1 (0-2)
**IUS**													
Levonorgestrel, 13.5[f]	98900	19.69±6.05	29.43±6.07	4 (3-5)	38.43	24.33±4.25	16.59	0.39	0.23	0.13	0.09	0.07	1 (0-2)
Levonorgestrel, 19.5[g]	40117	21.93±6.59	33.59±6.61	5 (3-5)	23.87	24.39±4.28	16.14	0.32	0.21	0.03	0.07	0.04	2 (1-2)
Levonorgestrel, 52[h]	2777293	31.18±8.87	35.91±7.60	4 (3-5)	11.46	24.37±4.27	18.71	1.19	0.68	1.72	0.06	0.37	2 (1-2)
**Injection**													
Medroxyprogesterone acetate	640529	33.30±9.31	35.60±8.41	3 (3-4)	21.12	24.85±4.92	32.74	1.71	0.76	2.16	0.08	0.67	2 (1-2)

[a]Education has been recorded as: 1) Pre-secondary education shorter than 9 years; 2) Pre-secondary education 9 years; 3) Secondary education; 4) Post-secondary education shorter than two years; 5) Post-secondary education two years or longer; 6) Research training; and 9) Unknown

[b]Have been calculated for the sub-group of gravid women

[c]Norplant 36 mg levonorgestrel for 5 years; [d]Implanon & Nexplanon: 68 mg etonogestrel for 3 years; [e]Jadelle 75 mg levonorgestrel for 5 years

[f]Jaydess 13.5 mg levonorgestrel for 3 years; [g]Kyleena 19.5 mg levonorgestrel for 5 years; [h]Mirena & Levosert 52 mg (20 microgram/24h) levonorgestrel for 8 and 6 years, respectively.

The percentage of nulligravid women was calculated by dividing the number of women with no recorded pregnancy in the Medical Birth Register in each category by the total number of women in that category. The percentage of smokers was calculated through dividing the number of smokers in each category by the number of women with history of pregnancy in that category. Other percentages were calculated by dividing the number of women with the characteristic of interest in each category by the total number of women in that category.

Abbreviations: HC, Hormonal Contraceptives; IUS, Intrauterine System; PCOS, polycystic ovary syndrome; BMI, Body Mass Index

© 2025 Hadizadeh F et al. *JAMA Oncol*.

eTable 4. Adjusted absolute risk, risk difference and crude incidence rates in users and never users of hormonal contraceptives (HC)

**Category**	**Adjusted absolute risk in never users***	**Adjusted absolute risk in users**	**Adjusted Risk Difference (95% CI)**	**Adjusted number needed to harm (95% CI)****	**Crude incidence rates in never users*****	**Crude incidence rates in users*****
Any type of HC	53.3	66.2	**13 (7 to 19)**	7752 (5350-14070)	91	69
Any type of Combined HC	57.7	64.7	**7 (0.6 to 13)**	14417 (7538-164745)	99	40
Any type of Progestin-only HC	55.6	67.3	**12 (5.5-18)**	8572 (5608 - 18174)	71	89
**Oral combined**						
Desogestrel	59.8	71.3	11 (-1 to 23)	8699	78	68
Levonorgestrel	59.1	64.5	5 (-4 to 14)	18522	89	34
Dienogest	60.2	62.8	3 (-14 to 20)	37934	78	49
Drospirenone	59.9	62.4	2 (-7 to 12)	40561	82	37
Nomegestrol	60.2	62.5	2 (-15 to 20)	43809	78	37
Norgestimate	60.2	61	1 (-12 to 14)	118031	81	19
Lynestrenol/Norethisterone	60.0	64.8	5 (-6 to 16)	20853	77	44
**Nonoral combined**						
Norelgestromin (Patch)	60.1	68.4	8 (-8 to 25)	12033	78	38
Etonogestrel (Vaginal ring)	60.0	63.2	3 (-8 to 14)	31312	80	36
**Oral Progestin-only**						
Desogestrel	57.9	68.2	**10 (1-19)**	9708 (5184 to 76195)	80	70
Lynestrenol	60.0	67.8	8 (-3-19)	12798	77	104
Norethisterone	60.0	62.4	2 (-8 to 13)	12587	78	88
**Nonoral Progestin-only**						
Etonogestrel (Implant)	59.5	72.4	**13 (1 to 25)**	7726 (4060 to 79783)	80	37
Levonorgestrel (Implant)	60.2	67.1	7 (-24 to 38)	14469	78	61
Levonorgestrel (IUS.13.5)	60.1	76.5	16 (-8 to 40)	6092	78	50
Levonorgestrel (IUS.19.5)	60.2	47.9	-12 (-34 to 9791)	-	78	52
Levonorgestrel (IUS.52)	59.1	67.2	8 (-8 to 17)	12303	70	128

© 2025 Hadizadeh F et al. *JAMA Oncol*.

Medroxyprogesterone acetate (Injection)	60.2	60.2	0 (-10 to 10)	-	77	108

*The adjusted absolute risk in never-users was estimated separately for each contraceptive type, controlling for the use of other types. This approach isolates the effect of never-use for each specific method while accounting for potential confounding from other contraceptive exposures.

** Confidence intervals for the number needed to harm (NNH) are not reported when the risk difference confidence interval includes negative values, as this implies potential benefit (i.e., number needed to treat) and renders the NNH confidence interval uninterpretable.

***Crude incidence rate is No. of events per 100,000 person-years

© 2025 Hadizadeh F et al. *JAMA Oncol*.

eTable 5. Duration of use analyses including all the progestin agents

	< 1 year	1 to <5 year	5 to 10 years	> 10 years
**Any type of hormonal contraceptives**				
HR (95% CI)	**1.11 (1.05-1.17)**	**1.21 (1.16-1.27)**	**1.34 (1.28-1.41)**	**1.33 (1.21-1.47)**
Number of events	1549	3626	2817	493
Person-years	2689851	6151480	3126504	389019
**Current users of any type of hormonal contraceptives**				
HR (95% CI)	0.93 (0.86-1.01)	**1.17 (1.12-1.23)**	**1.37 (1.30-1.44)**	**1.39 (1.25-1.54)**
Number of events	677	2685	2505	483
Person-years	1844552	5105678	2810507	377193
**Current+Recent users of any type of hormonal contraceptives**				
HR (95% CI)	1.04 (0.97-1.11)	**1.21 (1.15-1.26)**	**1.36 (1.30-1.44)**	**1.36 (1.23-1.51)**
Number of events	1070	3232	2788	493
Person-years	2273934	5750314	3089481	388998
**Main formulations**				
**Any type of Combined hormonal contraceptives**				
HR (95% CI)	1.05 (0.98-1.12)	**1.16 (1.09-1.23)**	**1.21 (1.09-1.35)**	**1.47 (1.08-2.00)**
Number of events	1086	1502	400	41
Person-years	2350634	3955913	1097361	81275
**Any type of Progestin-only hormonal contraceptives**				
HR (95% CI)	**1.09 (1.03-1.15)**	**1.19 (1.14-1.24)**	**1.32 (1.26-1.39)**	**1.30 (1.16-1.45)**
Number of events	1534	3074	2253	348
Person-years	2486369	3851311	1617876	188738
**Progestin agents**				
**Desogestrel-COC**				
HR (95% CI)	1.13 (9.72-1.31)	1.17 (1.02-1.35)	**1.48 (1.11-1.98)**	1.01 (0.25-4.03)
Number of events	173	210	46	2
Person-years	281533	304519	42319	2367
**Levonorgestrel-COC**				
HR (95% CI)	1.06 (0.97-1.15)	1.08 (1.00-1.17)	1.20 (1.03-1.40)	1.60 (1.03-2.49)
Number of events	608	681	167	20
Person-years	1635219	2179394	487014	37919
**Dienogest- COC**				
HR (95% CI)	1.15 (0.87-1.53)	0.89 (0.57-1.40)	1.36 (0.44-4.22)	NA
Number of events	49	19	3	0
Person-years	92690	47574	3780	15
**Drospirenone-COC**				
HR (95% CI)	0.99 (0.89-1.11)	1.11 (0.99-1.24)	1.16 (0.91-1.47)	1.06 (0.40-2.83)

© 2025 Hadizadeh F et al. *JAMA Oncol*.

Number of events	366	344	69	4
Person-years	995031	948979	156158	7095
**Nomegestrol-COC**				
HR (95% CI)	1.28 (0.97-1.69)	0.76(0.48-1.22)	NA	NA
Number of events	51	18	0	0
Person-years	112998	67350	3906	0
**Norgestimate-COC**				
HR (95% CI)	0.97 (0.77-1.22)	1.03 (0.82-1.30)	1.36 (0.83-2.22)	NA
Number of events	77	74	16	0
Person-years	408411	426995	62392	1076
**Lynestrenol/Norethisterone-COC**				
HR (95% CI)	1.10 (0.93-1.28)	1.04 (0.87-1.22)	1.10 (0.78-1.56)	1.48 (0.48-4.59)
Number of events	153	154	33	3
Person-years	361573	350564	59553	3373
**Norelgestromin-patch**				
HR (95% CI)	1.23 (0.97-1.54)	0.97 (0.64-1.48)	1.57 (0.51-4.86)	NA
Number of events	74	22	3	0
Person-years	195677	64230	3286	25
**Etonogestrel-Vaginal ring**				
HR (95% CI)	1.08 (0.95-1.22)	1.03 (0.84-1.24)	1.55 (0.92-2.62)	NA
Number of events	255	105	14	0
Person-years	702118	327629	16972	26
**Desogestrel-POP**				
HR (95% CI)	**1.08 (1.02-1.14)**	**1.23 (1.16-1.31)**	**1.49 (1.36-1.65)**	1.34 (1.03-1.74)
Number of events	1589	1150	452	58
Person-years	2569698	1663191	357790	32466
**Lynestrenol-POP**				
HR (95% CI)	1.08 (0.96-1.21)	1.13 (0.98-1.39)	1.35 (1.04-1.76)	1.04 (0.47-2.33)
Number of events	304	182	58	6
Person-years	330832	163689	31707	3422
**Norethisterone-POP**				
HR (95% CI)	0.92 (0.82-1.04)	**1.20 (1.03-1.38)**	1.08 (0.79-1.48)	**2.45 (1.35-4.44)**
Number of events	259	191	40	11
Person-years	357818	180180	30145	2793
**Etonogestrel-Implant**				
HR (95% CI)	1.07 (0.81-1.40)	**1.23 (1.09-1.39)**	**1.45 (1.17-1.80)**	1.15 (0.48-2.77)
Number of events	52	280	86	5
Person-years	232207	765226	142760	5414
**Levonorgestrel-Implant**				

© 2025 Hadizadeh F et al. *JAMA Oncol*.

HR (95% CI)	0.54 (0.08-3.86)	1.05 (0.56-1.95)	1.42 (0.74-2.73)	NA
Number of events	1	10	9	0
Person-years	5442	19933	7317	0
**Levonorgestrel-IUS 52 mg**				
HR (95% CI)	1.06 (0.96-1.17)	**1.14 (1.08-1.20)**	**1.21 (1.14-1.28)**	1.18 (1.01-1.38)
Number of events	431	1664	1309	163
Person-years	485621	1455327	755315	81029
**Levonorgestrel-IUS 13.5 mg**				
HR (95% CI)	1.51 (0.95-2.40)	1.18 (0.83-1.69)	NA	NA
Number of events	18	31	0	0
Person-years	37229	61671	0	0
**Levonorgestrel-IUS 19.5 mg**				
HR (95% CI)	0.75 (0.45-1.24)	0.94 (0.42-2.10)	NA	NA
Number of events	15	6	0	0
Person-years	30837	9280	0	0
**Medroxyprogesterone acetate-Injection**				
HR (95% CI)	0.95 (0.84-1.09)	0.95 (0.85-1.07)	1.15 (0.97-1.36)	1.16 (0.78-1.73)
Number of events	239	292	137	25
Person-years	265075	273167	86584	15703

All the analyses are adjusted for birth year, HC use in 2005, endometriosis, unilateral oophorectomy, hysterectomy, PCOS, sterilization, childbirth, and education.
Abbreviations. COC, Combined oral contraceptive; POP, Progestin-only pill; IUS, Intrauterine system
Statistically significant results after false discovery rate (FDR) correction are shown in bold.

© 2025 Hadizadeh F et al. *JAMA Oncol*.

eTable 6. Sensitivity analyses for ever versus never users of different hormonal contraceptives (HC)

**Category**			**Crude**	**Main**	**All sources cancer**		**Parous women**		**Main**	**Extended model**
	P.Y.	events	HR (95% CI)	HR (95% CI)	events	HR (95% CI)	P.Y.	events	HR (95% CI)	HR (95% CI)
Any Types of HC	2356854	8485	**1.23 (1.20 to 1.27)**	**1.24 (1.20 to 1.28)**	8925	**1.22 (1.18 to 1.26)**	8649483	6621	**1.25 (1.21 to 1.30)**	**1.25 (1.20 to 1.30)**
Any Combined	7485184	3029	**1.12 (1.07 to 1.17)**	**1.12 (1.07 to 1.17)**	3229	**1.10 (1.05 to 1.14)**	4898874	2206	**1.10 (1.04 to 1.15)**	**1.06 (1.003 to 1.11)**
Any Progestin-only	8144294	7209	**1.20 (1.16 to 1.24)**	**1.21 (1.17 to 1.25)**	7558	**1.20 (1.16 to 1.24)**	6089155	5803	**1.23 (1.18 to 1.27)**	**1.23 (1.18 to 1.28)**
**Combined oral**										
Desogestrel	630738	431	**1.19 (1.09** to **1.32)**	**1.19 (1.08** to **1.31)**	447	**1.16 (1.05** to **1.27)**	453884	289	1.10 (0.98-1.24)	1.07 (0.95 to 1.20)
Levonorgestrel	4339546	1476	**1.08 (1.02** to **1.15)**	**1.09 (1.03** to **1.15)**	1581	**1.07 (1.02** to **1.13)**	2718871	1083	**1.09 (1.02-1.16)**	1.06 (0.99 to 1.14)
Dienogest	144059	71	1.04 (0.82 to 1.32)	1.05 (0.83 to 1.33)	77	1.07 (0.86 to 1.35)	84354	50	0.98 (0.74-1.30)	0.97 (0.73 to 1.28)
Drospirenone	2107262	783	1.04 (0.97 to 1.12)	1.04 (0.96 to 1.12)	843	1.04 (0.96 to 1.12)	1339271	576	1.05 (0.96-1.14)	1.03 (0.94 to 1.12)
Nomegestrol	184254	69	1.03 (0.81 to 1.31)	1.04 (0.82 to 1.32)	74	1.04 (0.82 to 1.31)	103977	51	1.04 (0.79-1.37)	1.02 (0.77 to 1.35)
Norgestimate	898874	167	1.01 (0.87 to 1.18)	1.01 (0.87 to 1.18)	187	1.01 (0.87 to 1.17)	534645	133	1.16 (0.98-1.39)	1.14 (0.96 to 1.35)
Norethisterone/ Lynestrenol	775063	343	1.08 (0.97 to 1.20)	1.08 (0.96 to 1.20)	364	1.06 (0.95 to 1.18)	536722	254	1.08 (0.95-1.22)	1.05 (0.92 to 1.19)
**Combined non oral**										
Norelgestromin (Patch)	263218	99	1.11 (0.91 to 1.36)	1.15 (0.94 to 1.40)	105	1.12 (0.93 to 1.36)	182058	72	1.08 (0.86-1.37)	1.07 (0.85 to 1.36)
Etonogestrel (Vaginal ring)	1046745	374	1.06 (0.95 to 1.18)	1.05 (0.95 to 1.17)	394	1.02 (0.92 to 1.13)	695439	274	1.02 (0.90-1.15)	0.99 (0.88 to 1.12)

© 2025 Hadizadeh F et al. *JAMA Oncol*.

**Progestin-only oral**										
Desogestrel	4623146	3249	**1.17 (1.12 to 1.22)**	**1.18 (1.13 to 1.23)**	3411	**1.17 (1.12 to 1.22)**	3359497	2564	**1.19 (1.13-1.24**	**1.17 (1.11 to 1.22)**
Levonorgestrel	80	0	NA	NA	NA	NA	80	0	NA	NA
Lynestrenol	529651	550	**1.13 (1.04 to 1.23)**	**1.13 (1.04 to 1.23)**	567	**1.11 (1.02 to 1.21)**	420078	444	**1.16 (1.05-1.27**	**1.13 (1.03 to 1.25)**
Norethisterone	570936	501	1.04 (0.95 to 1.13)	1.04 (0.95 to 1.14)	522	1.03 (0.95 to 1.13)	449297	393	1.03 (0.93-1.14)	1.01 (0.91 to 1.12)
**Progestin-only non oral**										
Implant										
Levonorgestrel.36	235	1	3.80 (0.54 to 27)	3.92 (0.55 to 27.8)	1	3.70 (0.52 to 26.2)	201	0	NA	NA
Etonogestrel.68	1145607	423	**1.18 (1.07 to 1.30)**	**1.22 (1.11 to 1.35)**	467	**1.25 (1.13 to 1.37)**	741399	311	**1.19 (1.06-1.33)**	**1.19 (1.07 to 1.34)**
Levonorgestrel.75	32692	20	1.10 (0.71 to 1.70)	1.12 (0.72 to 1.73)	21	1.10 (0.71 to 1.68)	23310	16	1.18 (0.72-1.92)	1.19 (0.73 to 1.94)
IUS										
Levonorgestrel .13.5	98900	49	1.27 (0.96 to 1.68)	1.28 (0.96 to 1.69)	49	1.19 (0.90 to 1.58)	58661	40	1.33 (0.97-1.81)	1.31 (0.96 to 1.80)
Levonorgestrel .19.5	40117	21	0.78 (0.51 to 1.20)	0.79 (0.52 to 1.22)	22	0.79 (0.52 to 1.21)	29144	18	0.77 (0.49-1.23)	0.77 (0.48 to 1.22)
Levonorgestrel.52	2777293	3567	**1.14 (1.10 to 1.18)**	**1.13 (1.09 to 1.18)**	373	**1.14 (1.09 to 1.18)**	2331101	3075	**1.15 (1.10-1.20)**	**1.15 (1.10 to 1.20)**
Injection										
Medroxyprogesterone acetate	640326	693	0.96 (0.89 to 1.03)	1.00 (0.93 to 1.08)	725	1.00 (0.93 to 1.08)	453784	510	1.02 (0.94-1.12)	1.03 (0.94 to 1.13)

Crude analyses include only the main exposure(s)

© 2025 Hadizadeh F et al. *JAMA Oncol*.

Main, all sources cancer and main parous analyses include adjustment for all the main covariates (birth year, endometriosis, unilateral oophorectomy, hysterectomy, PCOS, sterilization, childbirth, and education)
All sources cancer: This refers to analyses where the definition of breast cancer based on the Cancer Register was supplemented with additional cases identified in the patient Register using ICD-10 codes “C50” and “D05”.
Parous Main: This analysis included the same covariates as the main model but was restricted to parous women and the Parous Extended includes also age at first birth, prior use of hormonal contraceptives, body mass index (BMI), and smoking status, in addition to the main covariates, with data available exclusively from the Medical Birth Register for the subset of women with history of pregnancy.

Abbreviations: P.Y., Person Year; HR, Hazard Ratio; CI, Confidence Interval; HC, Hormonal Contraceptives; IUS, Intrauterine System

© 2025 Hadizadeh F et al. *JAMA Oncol*.

eTable 7. Sensitivity analyses for duration of use analyses of different hormonal contraceptives

		Crude	Main	365 days limited	All sources cancer	Parous Main	Parous Extended
**Any type of hormonal contraceptive**							
< 1 year	HR (95% CI)	1.10 (1.04 t0 1.17)	1.11 (1.05-1.17)	1.11 (1.04 to 1.17)	1.10 (1.04 to 1.16)	1.13 (1.06 to 1.20)	1.11 (1.04 to 1.19)
	Number of events	1549			1646	1164	
	Person-years	2689851				1908602	
1 to <5 year	HR (95% CI)	1.22 (1.17 to 1.27)	1.21 (1.16-1.27)	1.21 (1.16 to 1.27)	1.20 (1.15 to 1.25)	1.23 (1.17 to 1.29)	1.22 (1.16 to 1.28)
	Number of events	3626			3844	2805	
	Person-years	6151480				4311918	
5 to 10 years	HR (95% CI)	1.32 (1.27 to 1.38)	1.34 (1.28-1.41)	1.36 (1.29 to 1.43)	1.32 (1.26 to 1.39)	1.33 (1.25 to 1.41)	1.34 (1.26 to 1.42)
	Number of events	2817			2928	2256	
	Person-years	3126504				2179521	
> 10 years	HR (95% CI)	1.29 (1.18 to 1.41)	1.33 (1.21-1.47)	1.26 (1.13 to 1.41)	1.31 (1.19 to 1.45)	1.32 (1.18 to 1.48)	1.37 (1.22 to 1.53)
	Number of events	493			507	396	
	Person-years	389019				249441	
**Any type of Combined hormonal contraceptive**							
< 1 year	HR (95% CI)	1.05 (0.98 to 1.12)	1.05 (0.98-1.12)	1.05 (0.98 to 1.12)	1.05 (0.99 to 1.12)	1.03 (0.96 to 1.11)	1.00 (0.93 to 1.08)
	Number of events	1086			1170	818	
	Person-years	2350634				1633682	
1 to <5 year	HR (95% CI)	1.17 (1.10 to 1.23)	1.16 (1.09-1.23)	1.15 (1.08 to 1.22)	1.13 (1.07 to 1.20)	1.14 (1.06 to 1.22)	1.10 (1.02 to 1.18)
	Number of events	1502			1598	1104	
	Person-years	3955913				2595044	
5 to 10 years	HR (95% CI)	1.20 (1.08 to 1.33)	1.21 (1.09-1.35)	1.27 (1.14 to 1.42)	1.16 (1.05 to 1.29)	1.14 (1.01 to 1.30)	1.12 (0.98 to 1.27)
	Number of events	400			417	261	
	Person-years	1097361				637345	
> 10 years	HR (95% CI)	1.41 (1.03 to 1.91)	1.47 (1.08-2.00)	1.70 (1.14 to 2.55)	1.46 (1.08 to 1.96)	1.48 (0.98 to 2.24)	1.48 (0.98 to 2.24)
	Number of events	41			44	23	
	Person-years	81275				32802	
**Any type of Progestin-only hormonal contraceptive**							
< 1 year	HR (95% CI)	1.08 (1.02 to 1.14)	1.09 (1.04-1.16)	1.09 (1.03 to 1.15)	1.08 (1.03 to 1.14)	1.12 (1.05 to 1.19)	1.10 (1.03 to 1.17)
	Number of events	1534			1625	1203	

© 2025 Hadizadeh F et al. *JAMA Oncol*.

	Person-years	2486369				1853149	
**1 to <5 year**	HR (95% CI)	1.19 (1.14 to 1.24)	1.20 (1.15-1.25)	1.20 (1.15 to 1.25)	1.19 (1.14 to 1.24)	1.20 (1.14 to 1.26)	1.21 (1.15 to 1.27)
	Number of events	3074			3242	2447	
	Person-years	3851311				2851851	
**5 to 10 years**	HR (95% CI)	1.30 (1.24 to 1.36)	1.34 (1.27-1.40)	1.32 (1.26 to 1.40)	1.31 (1.25 to 1.38)	1.31 (1.24 to 1.39)	1.34 (1.26 to 1.42)
	Number of events	2253			2337	1860	
	Person-years	1617876				1238797	
**> 10 years**	HR (95% CI)	1.25 (1.12 to 1.39)	1.32 (1.18-1.47)	1.24 (1.10 to 1.41)	1.27 (1.14 to 1.42)	1.28 (1.13 to 1.45)	1.33 (1.17 to 1.51)
	Number of events	348			534	293	
	Person-years	188738				145358	

Crude analyses include only the main exposure(s)

Main, all sources cancer and main parous analyses include adjustment for all the main covariates (birth year, HC use in 2005, endometriosis, unilateral oophorectomy, hysterectomy, PCOS, sterilization, childbirth, and education).

365 days limited in cases where the calculated AMD based on the Prescribed Drug Register data exceeded the number of days in a year, a maximum limit of 365 days per year was applied to represent full exposure to HC, and the values above 365 days were rounded down to 365 days. Sensitivity analyses were conducted to assess the impact of limiting the number of days of using HC to 365 days per year (365 days limited analyses) on the estimated effect.

All sources cancer: This refers to analyses where the definition of breast cancer based on the Cancer Register was supplemented with additional cases identified in the Inpatient Register using ICD-10 codes “C50” and “D05”.

Parous Main: This analysis included the same covariates as the main model but was restricted to parous women and the Parous Extended model includes age at first birth, prior use of hormonal contraceptives, body mass index (BMI), and smoking status, in addition to the main covariates, with data available exclusively from the Medical Birth Register for the subset of women with history of pregnancy.

Abbreviations: HR, Hazard Ratio; CI, Confidence Interval

© 2025 Hadizadeh F et al. *JAMA Oncol*.

eTable 8. Schoenfeld residual p-values for all the main exposures and study covariates.

Category	chisq	Schoenfeld test p-value*
Any type of hormonal contraceptive	0.1254	0.7231
Any type of combined	0.6711	0.4127
Any type of progestin-only	0.2953	0.5868
**Oral combined**		
Desogestrel	3.38	0.0661
Levonorgestrel	2.35	0.1253
Dienogest	1.01	0.3142
Drospirenone	0.401	0.5265
Nomegestrol	0.04.75	0.8275
Norgestimate	0.351	0.5534
Lynestrenol/Norethisterone	0.314	0.5753
**Non oral combined**		
Norelgestromin (Patch)	2.75	0.0971
Etonogestrel (Vaginal ring)	1.53	0.2163
**Oral Progestin-only**		
Desogestrel	1.07	0.3012
Lynestrenol	0.376	0.5399
Norethisterone	0.0024	0.9606
**Non oral Progestin-only**		
Etonogestrel (Implant)	5.48	0.0192
Levonorgestrel (Implant)	0.499	0.4798
Levonorgestrel (IUS 13.5)	0.0316	0.8589
Levonorgestrel (IUS 19.5)	1.58	0.2081
Levonorgestrel (IUS 52)	4.29	0.0383
Medroxyprogesterone acetate (Injection)	0.210	0.6471
**Other study covariates**		
Birth_Year	0.168	4.1e-05*
Endometriosis	0.535	0.4644
Uni_OOph	1.34	0.2475
Hysterectomy	0.876	0.3494
PCOS	1.00	0.3164
Childbirth**	2.16	0.1419
Education	7.25	0.2984
Sterilization	0.589	0.4427

* Birth year was significant after FDR correction for multiple testing (q value: 0.001) and was further investigated in Supplementary eFigure 4 and eMethods. None of the other p-values were significant after FDR correction.

** To enhance model convergence and feasibility, the variable "childbirth" was coded as a numeric variable in this specific analysis. In the main model, however, it was treated as a categorical factor. This deviation was necessary because modelling "childbirth" as a factor led to convergence issues, likely due to the increased number of parameters introduced by multiple

© 2025 Hadizadeh F et al. *JAMA Oncol*.

levels and limited data per category. Treating it as numeric reduced model complexity and allowed the analysis to proceed.

© 2025 Hadizadeh F et al. *JAMA Oncol*.

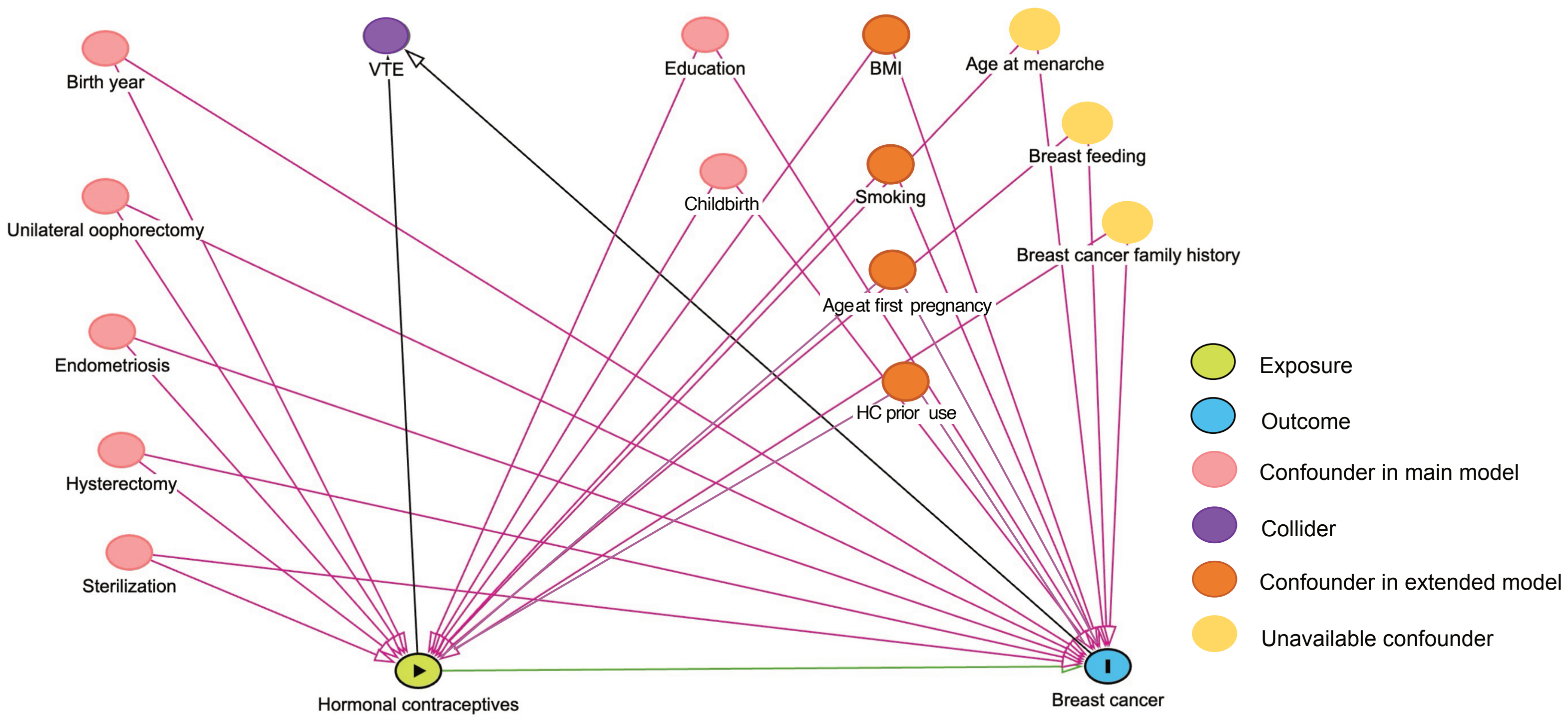

eFigure 1. Directed Acyclic Graph (DAG) on the association of hormonal contraceptive (HC) use and breast cancer.

© 2025 Hadizadeh F et al. *JAMA Oncol*.

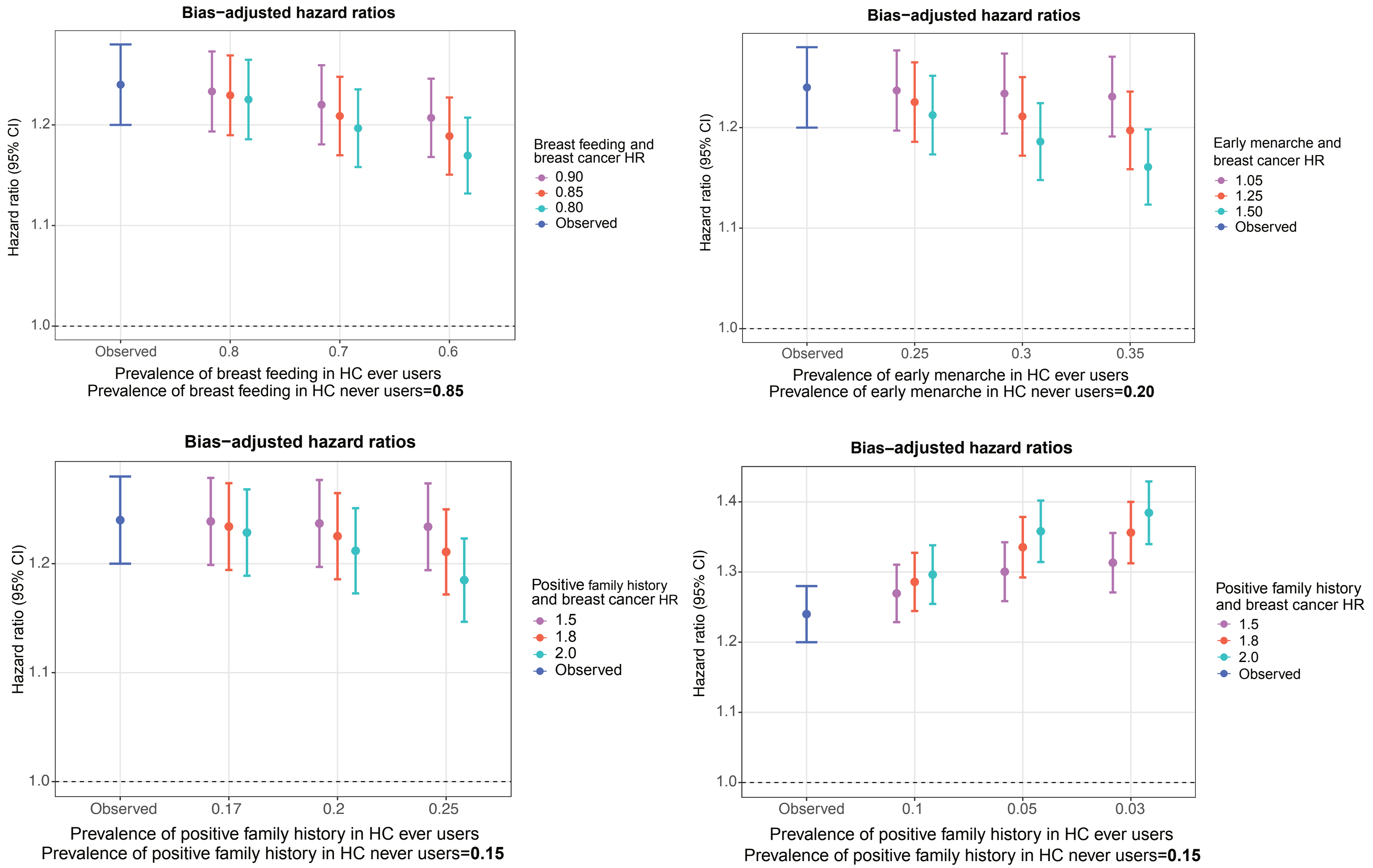

© 2025 Hadizadeh F et al. *JAMA Oncol*.

eFigure 2. Quantitative bias analysis investigating the impact of binary unmeasured confounders of breast feeding, early menarche and family history of breast cancer on the estimated breast cancer hazard ratios in users of hormonal contraceptives.

Prevalence of the confounder in the never user group was constant. Prevalence of the confounder in the hormonal contraceptive (HC) user group (X-axis) and the effect of the confounder on the outcome on the hazard ratio scale were varied. Observed hazard ratio is 1.24 (1.20-1.28) and Y-axis demonstrates the bias-adjusted hazard ratios for each scenario.
Regarding positive family history of breast cancer, current evidence suggests that women with a positive family history of breast cancer are less likely to use or be prescribed hormonal contraceptives due to the associated risk. However, in the study by Mørch et al.[8], the prevalence of family history was slightly higher among users than never-users. Therefore, we explored both scenarios in our analysis.
Abbreviations. HR, hazard ratio; CI, Confidence Interval

© 2025 Hadizadeh F et al. *JAMA Oncol*.

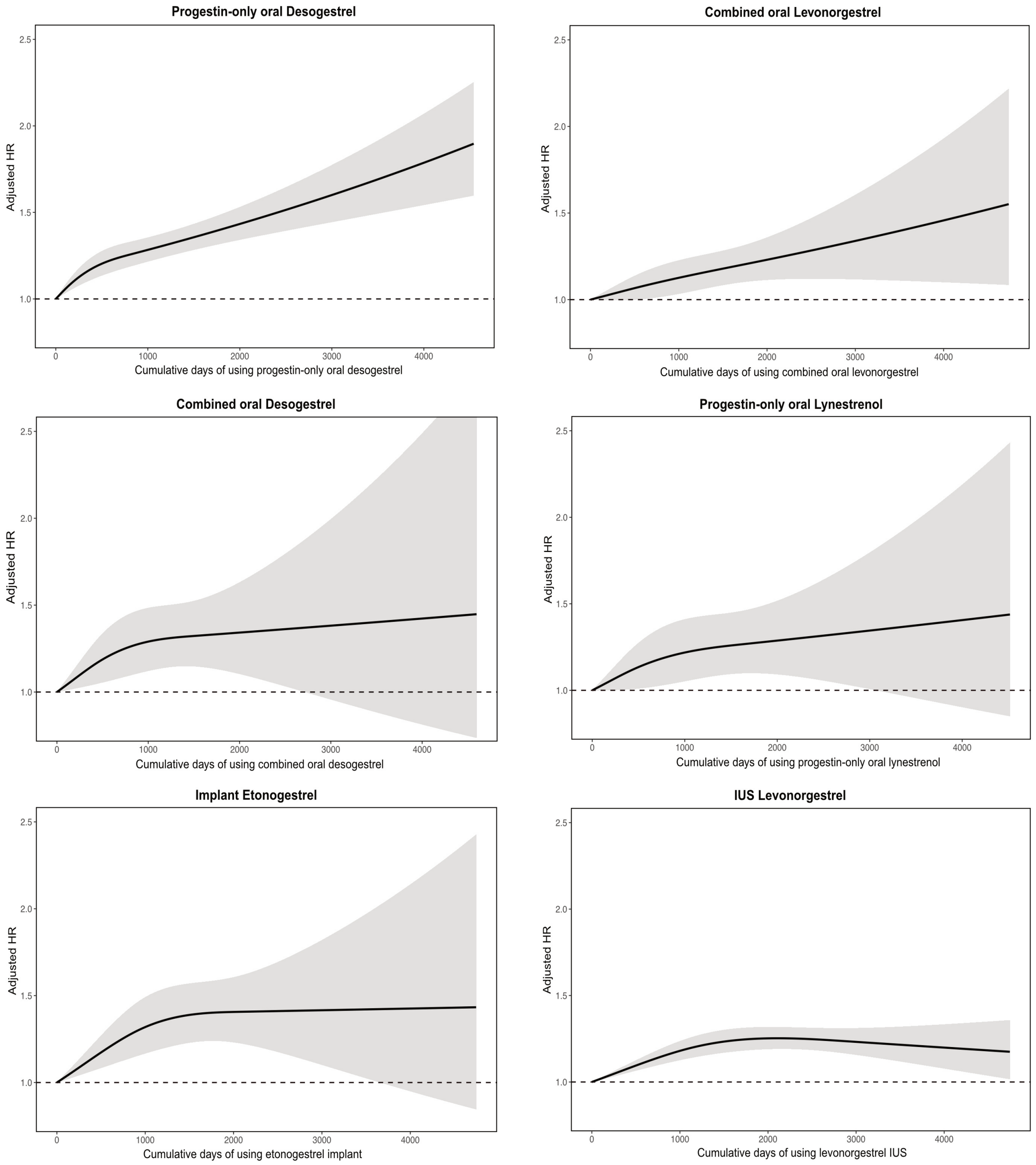

eFigure 3. Restricted cubic spline curves for different progestin agents.
Curves depict the nonlinear association between duration of exposure to different progestins and breast cancer risk, modeled by 3-knot restricted cubic splines using rcs() function of the "rms" R package. Only associations that were statistically significant in the ever versus never use analyses are illustrated.

© 2025 Hadizadeh F et al. *JAMA Oncol*.

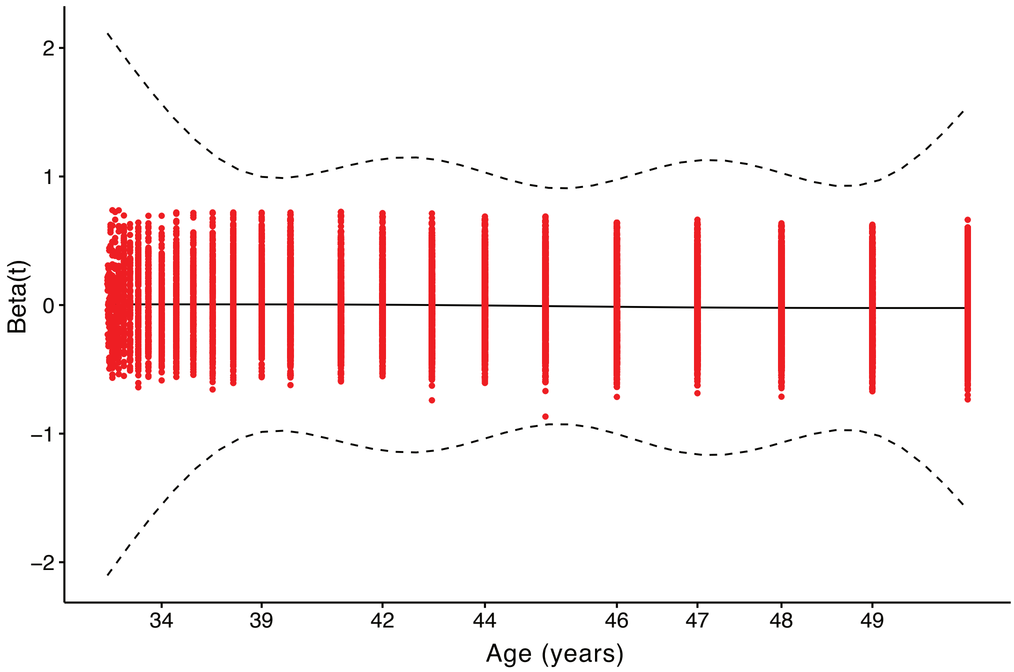

eFigure 4. Schoenfeld residual plot for covariate Birth year. Birth year was the only variable (covariate or exposure) which significantly violated the assumption of proportional hazards (FDR adjusted p-value <0.05). For information on other variables, see Supplementary eTable 8. Since age was used as the primary time-scale in our analyses, the X-axis in the plot is age (years)

© 2025 Hadizadeh F et al. *JAMA Oncol*.
