# Supplementary material for: Hormonal Contraceptive Formulations and Breast Cancer Risk in Adolescents and Premenopausal Women
Source: JAMA Oncol. 2025 Oct 30;11(12):1497–506. doi: 10.1001/jamaoncol.2025.4480 (PMC12576617; doi:10.1001/jamaoncol.2025.4480)
Supplement: Supplement 2. — Data Sharing Statement [file jamaoncol-e254480-s002.pdf]

# Data Sharing Statement

Hadizadeh. Hormonal Contraceptive Formulations and Breast Cancer Risk in Premenopausal Adolescents and Women. *JAMA Oncol*. Published October 30, 2025. doi:10.1001/jamaoncol.2025.4480

**Data**
**Data available:** No

**Additional Information**
**Explanation for why data not available:** Data from the national Swedish registers can be accessed by researchers affiliated with a Swedish research institute upon approval by the Swedish Ethical Authority
